# Supplementary figures and images for: A novel network security situation assessment model based on multiple strategies whale optimization algorithm and bidirectional GRU (part 2 of 2)
Source: PeerJ Comput Sci. 2023 Dec 12;9:e1729. doi: 10.7717/peerj-cs.1729 (PMC10773833; doi:10.7717/peerj-cs.1729)

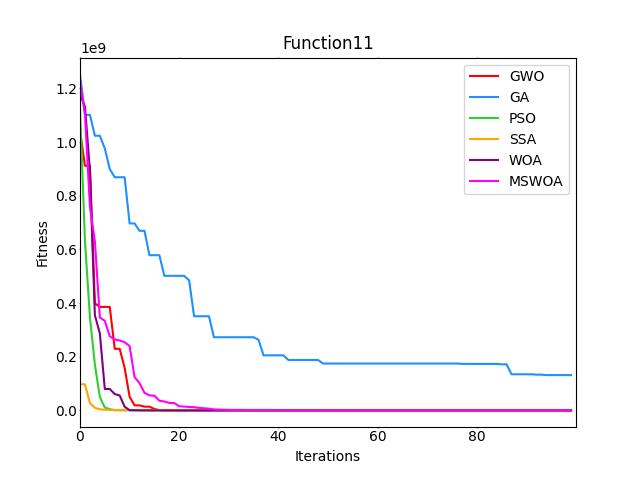

Supplement: Supplemental Information 1 [file peerj-cs-09-1729-s001.zip › code1/inteligent_algorithm_submit/Function11/func11/pic28.jpg]

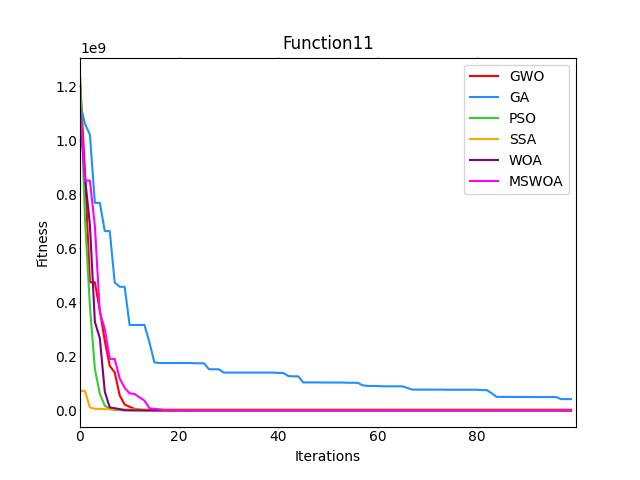

Supplement: Supplemental Information 1 [file peerj-cs-09-1729-s001.zip › code1/inteligent_algorithm_submit/Function11/func11/pic29.jpg]

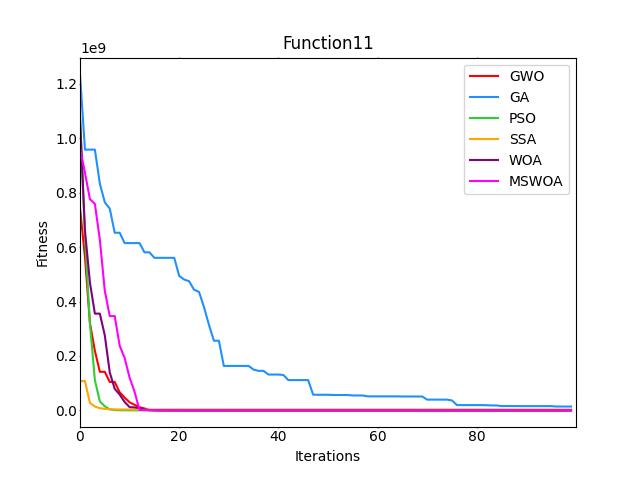

Supplement: Supplemental Information 1 [file peerj-cs-09-1729-s001.zip › code1/inteligent_algorithm_submit/Function11/func11/pic3.jpg]

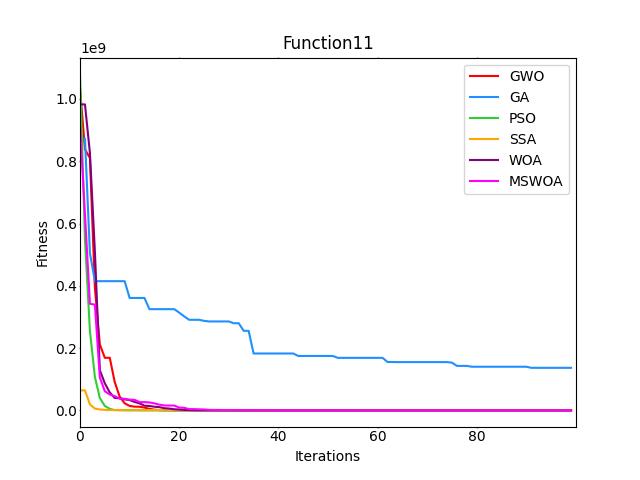

Supplement: Supplemental Information 1 [file peerj-cs-09-1729-s001.zip › code1/inteligent_algorithm_submit/Function11/func11/pic30.jpg]

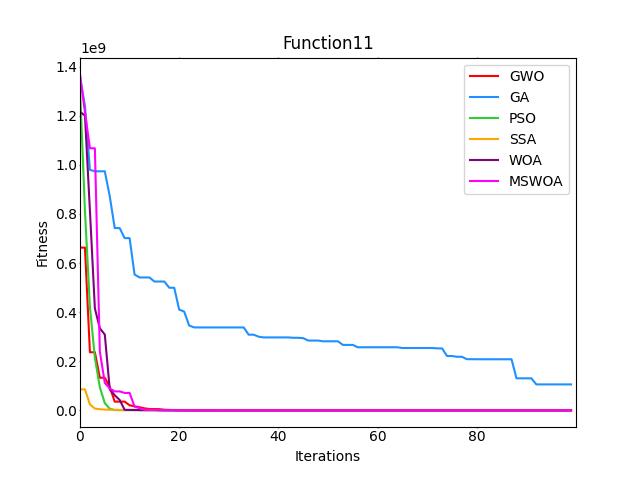

Supplement: Supplemental Information 1 [file peerj-cs-09-1729-s001.zip › code1/inteligent_algorithm_submit/Function11/func11/pic4.jpg]

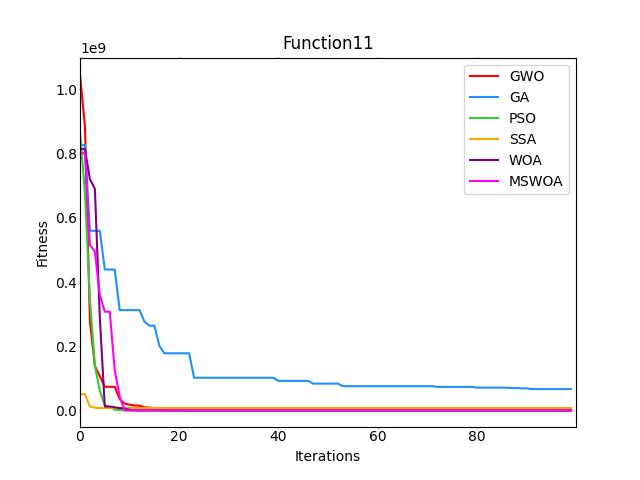

Supplement: Supplemental Information 1 [file peerj-cs-09-1729-s001.zip › code1/inteligent_algorithm_submit/Function11/func11/pic5.jpg]

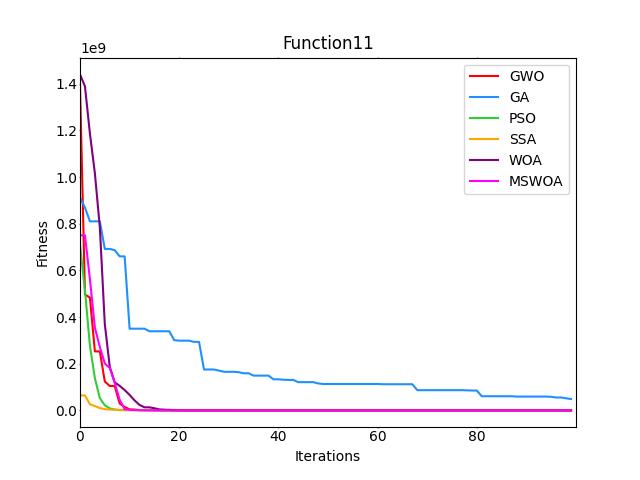

Supplement: Supplemental Information 1 [file peerj-cs-09-1729-s001.zip › code1/inteligent_algorithm_submit/Function11/func11/pic6.jpg]

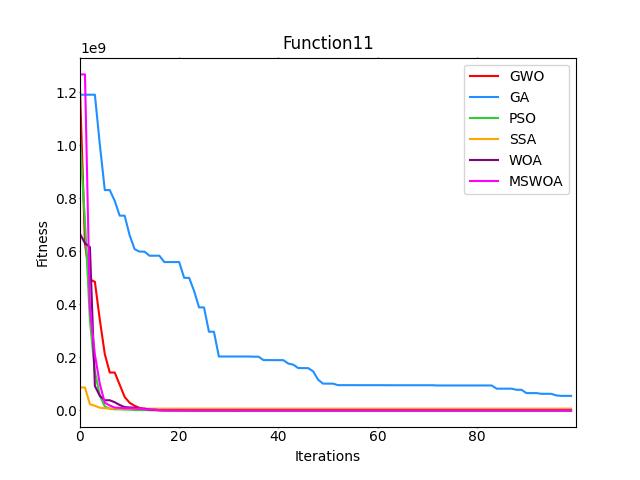

Supplement: Supplemental Information 1 [file peerj-cs-09-1729-s001.zip › code1/inteligent_algorithm_submit/Function11/func11/pic7.jpg]

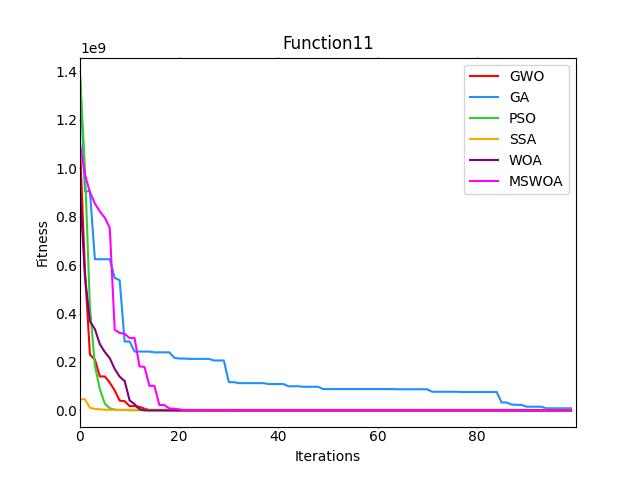

Supplement: Supplemental Information 1 [file peerj-cs-09-1729-s001.zip › code1/inteligent_algorithm_submit/Function11/func11/pic8.jpg]

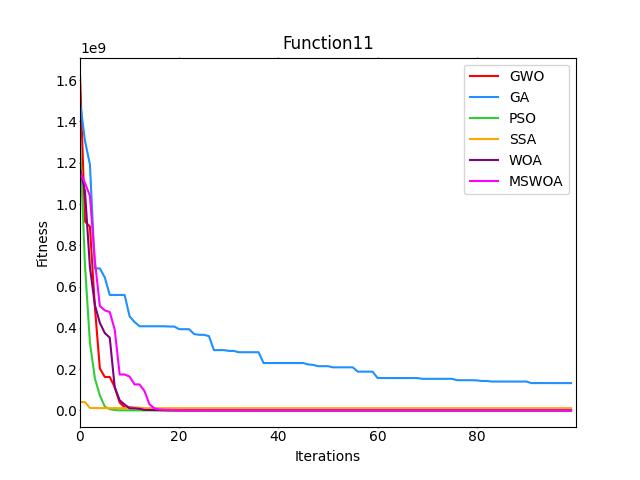

Supplement: Supplemental Information 1 [file peerj-cs-09-1729-s001.zip › code1/inteligent_algorithm_submit/Function11/func11/pic9.jpg]

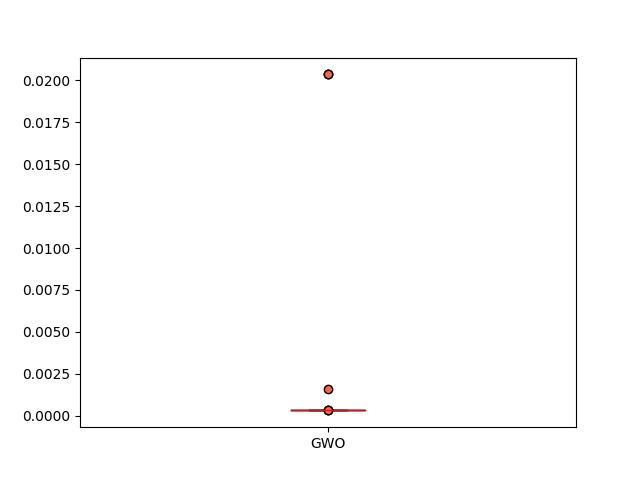

Supplement: Supplemental Information 1 [file peerj-cs-09-1729-s001.zip › code1/inteligent_algorithm_submit/Function12/boxplot_func12/pic1.jpg]

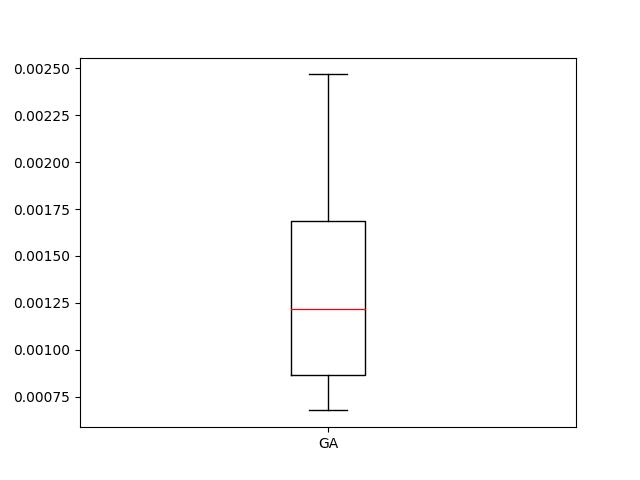

Supplement: Supplemental Information 1 [file peerj-cs-09-1729-s001.zip › code1/inteligent_algorithm_submit/Function12/boxplot_func12/pic2.jpg]

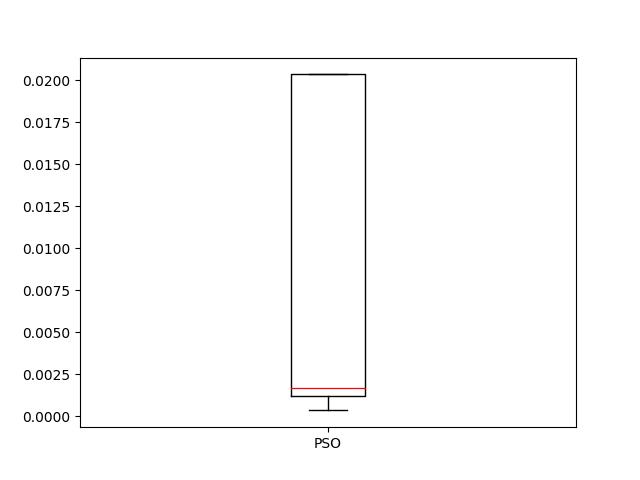

Supplement: Supplemental Information 1 [file peerj-cs-09-1729-s001.zip › code1/inteligent_algorithm_submit/Function12/boxplot_func12/pic3.jpg]

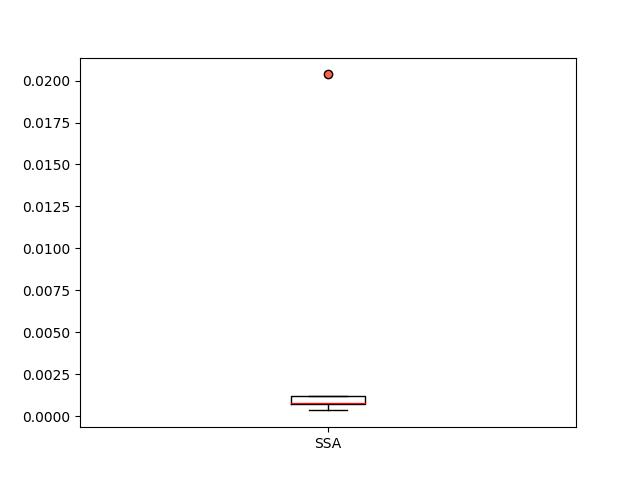

Supplement: Supplemental Information 1 [file peerj-cs-09-1729-s001.zip › code1/inteligent_algorithm_submit/Function12/boxplot_func12/pic4.jpg]

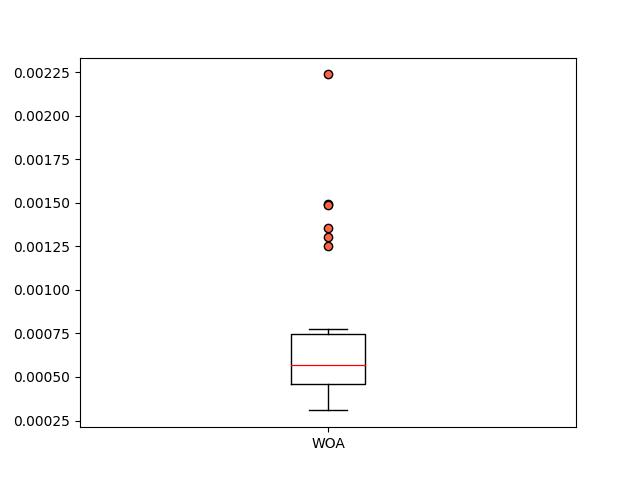

Supplement: Supplemental Information 1 [file peerj-cs-09-1729-s001.zip › code1/inteligent_algorithm_submit/Function12/boxplot_func12/pic5.jpg]

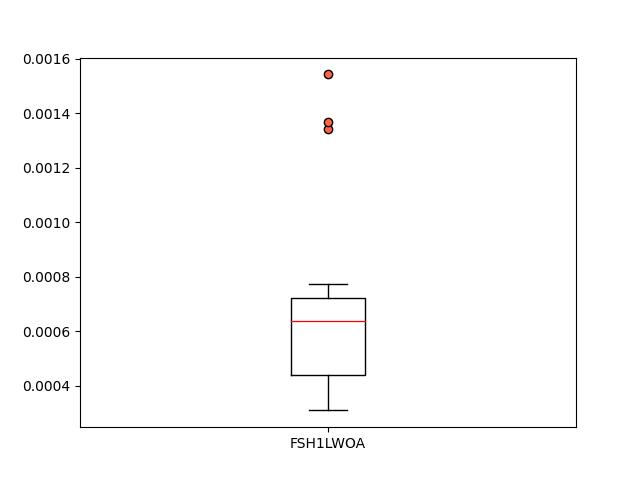

Supplement: Supplemental Information 1 [file peerj-cs-09-1729-s001.zip › code1/inteligent_algorithm_submit/Function12/boxplot_func12/pic6.jpg]

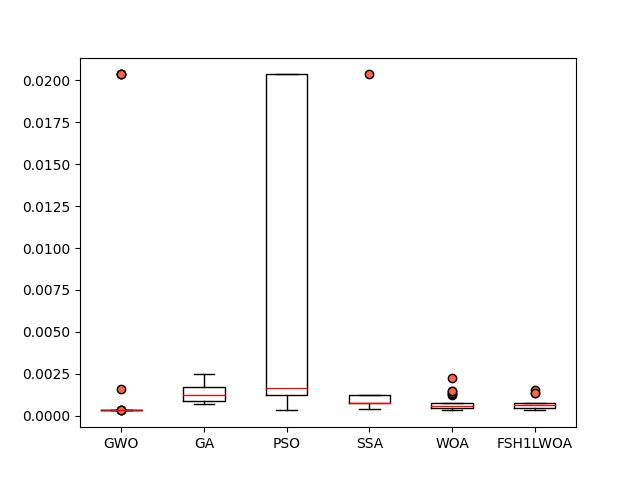

Supplement: Supplemental Information 1 [file peerj-cs-09-1729-s001.zip › code1/inteligent_algorithm_submit/Function12/boxplot_func12/pic_total.jpg]

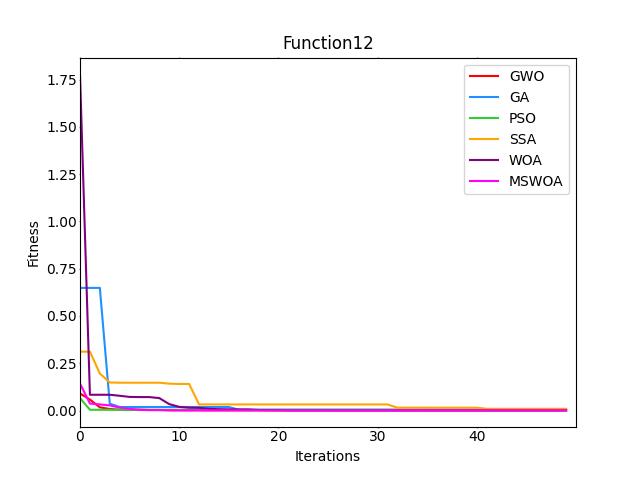

Supplement: Supplemental Information 1 [file peerj-cs-09-1729-s001.zip › code1/inteligent_algorithm_submit/Function12/func12/pic1.jpg]

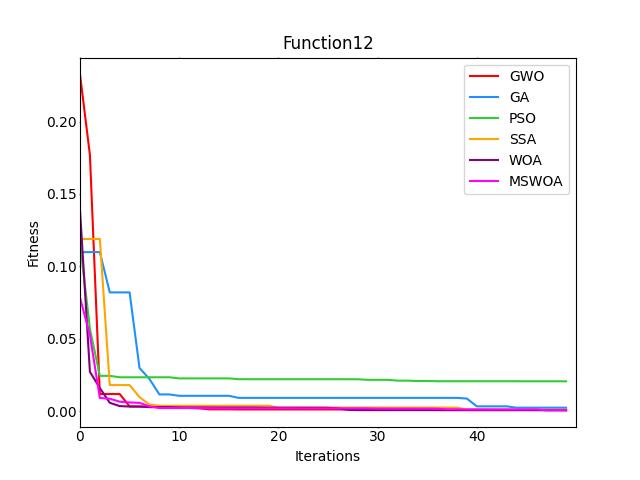

Supplement: Supplemental Information 1 [file peerj-cs-09-1729-s001.zip › code1/inteligent_algorithm_submit/Function12/func12/pic10.jpg]

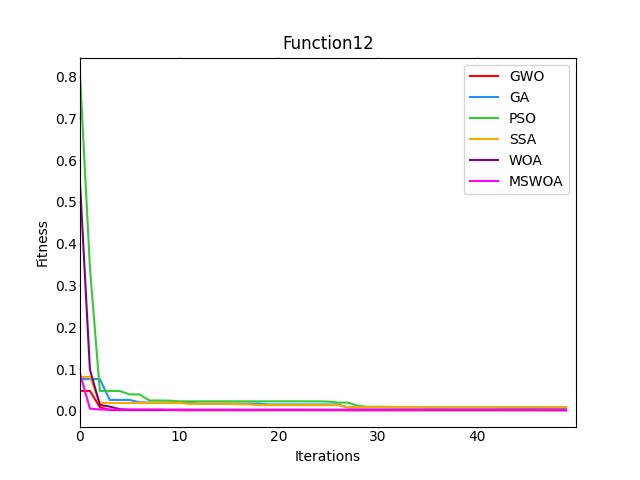

Supplement: Supplemental Information 1 [file peerj-cs-09-1729-s001.zip › code1/inteligent_algorithm_submit/Function12/func12/pic11.jpg]

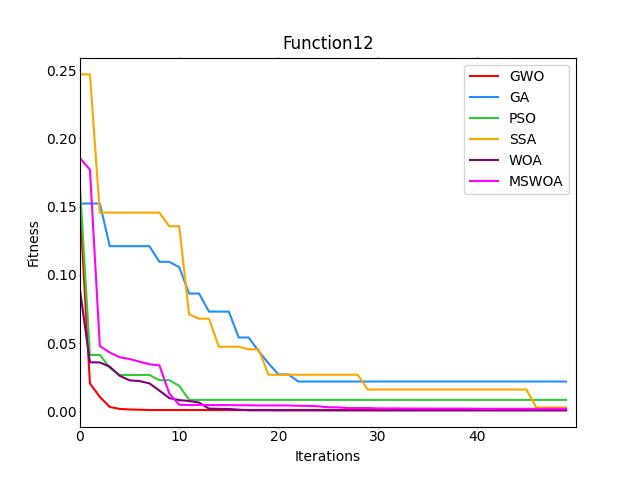

Supplement: Supplemental Information 1 [file peerj-cs-09-1729-s001.zip › code1/inteligent_algorithm_submit/Function12/func12/pic12.jpg]

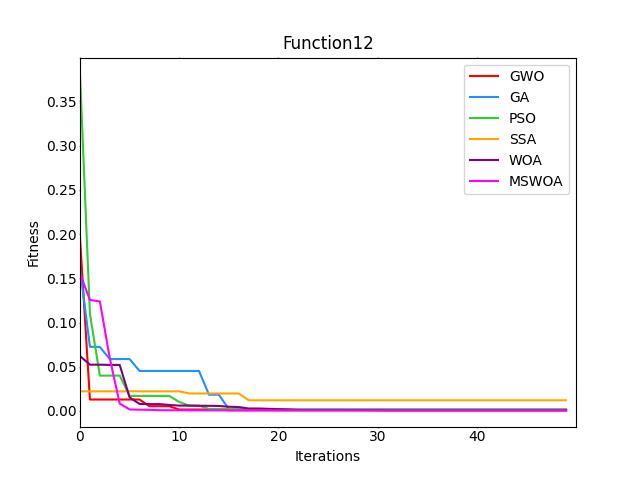

Supplement: Supplemental Information 1 [file peerj-cs-09-1729-s001.zip › code1/inteligent_algorithm_submit/Function12/func12/pic13.jpg]

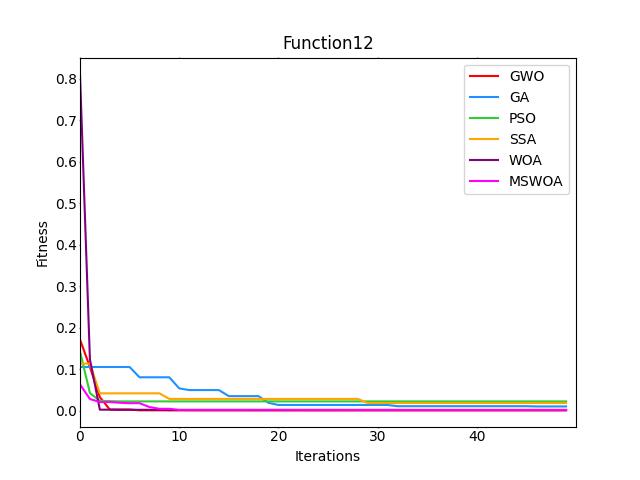

Supplement: Supplemental Information 1 [file peerj-cs-09-1729-s001.zip › code1/inteligent_algorithm_submit/Function12/func12/pic14.jpg]

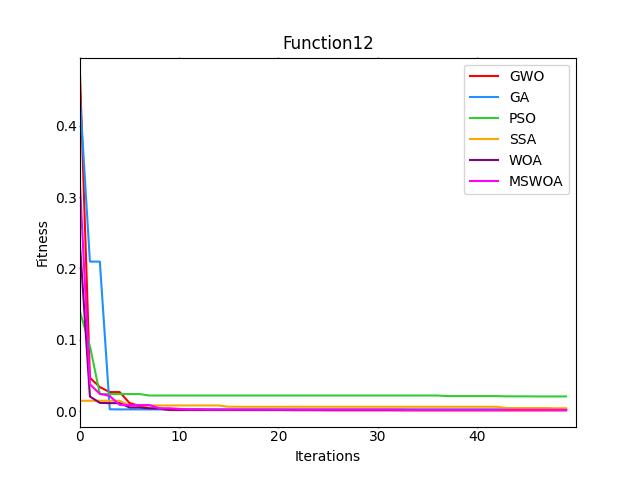

Supplement: Supplemental Information 1 [file peerj-cs-09-1729-s001.zip › code1/inteligent_algorithm_submit/Function12/func12/pic15.jpg]

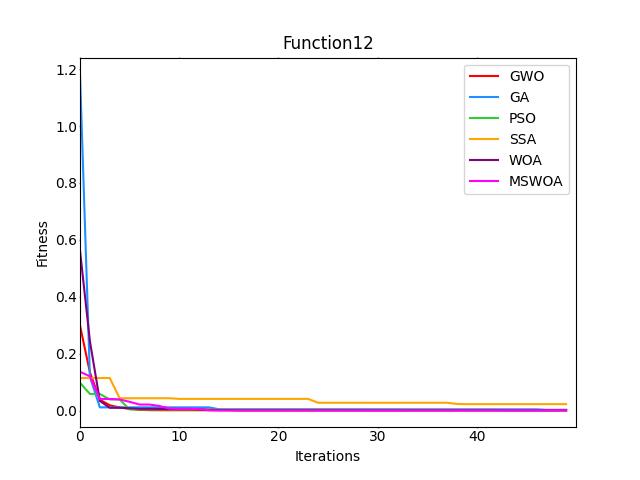

Supplement: Supplemental Information 1 [file peerj-cs-09-1729-s001.zip › code1/inteligent_algorithm_submit/Function12/func12/pic16.jpg]

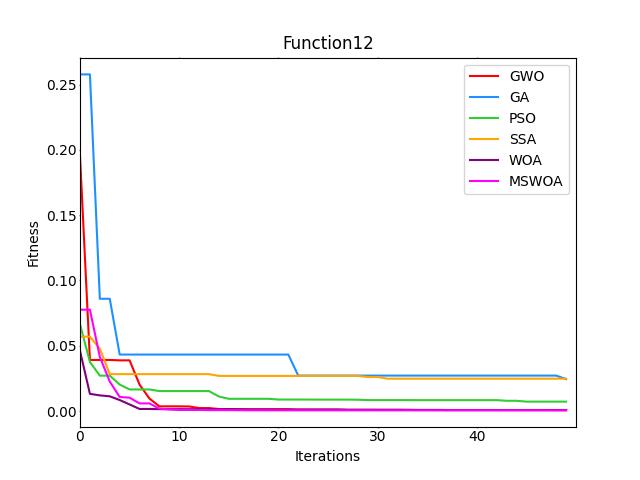

Supplement: Supplemental Information 1 [file peerj-cs-09-1729-s001.zip › code1/inteligent_algorithm_submit/Function12/func12/pic17.jpg]

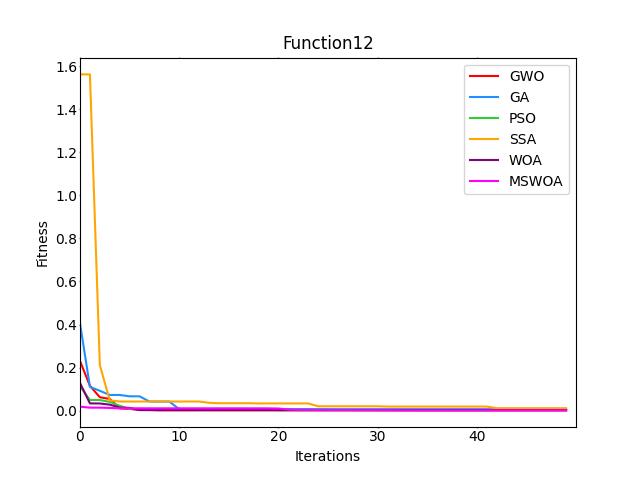

Supplement: Supplemental Information 1 [file peerj-cs-09-1729-s001.zip › code1/inteligent_algorithm_submit/Function12/func12/pic18.jpg]

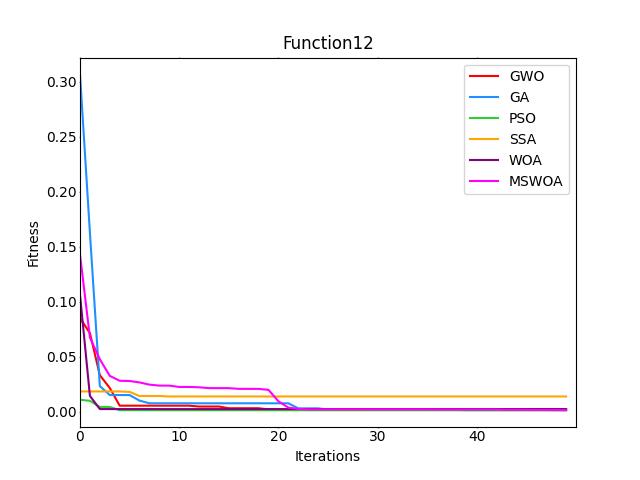

Supplement: Supplemental Information 1 [file peerj-cs-09-1729-s001.zip › code1/inteligent_algorithm_submit/Function12/func12/pic19.jpg]

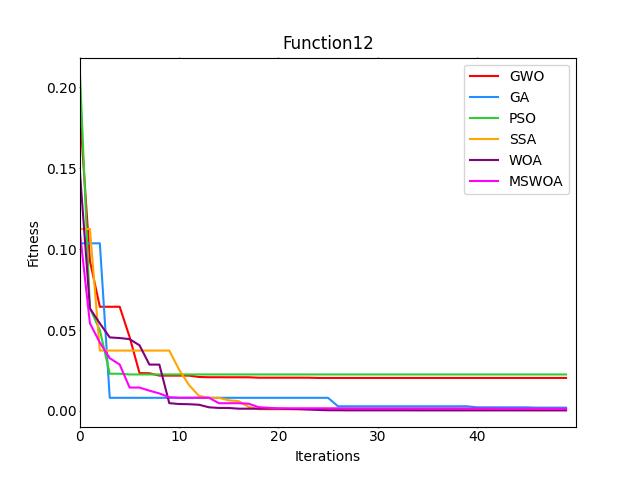

Supplement: Supplemental Information 1 [file peerj-cs-09-1729-s001.zip › code1/inteligent_algorithm_submit/Function12/func12/pic2.jpg]

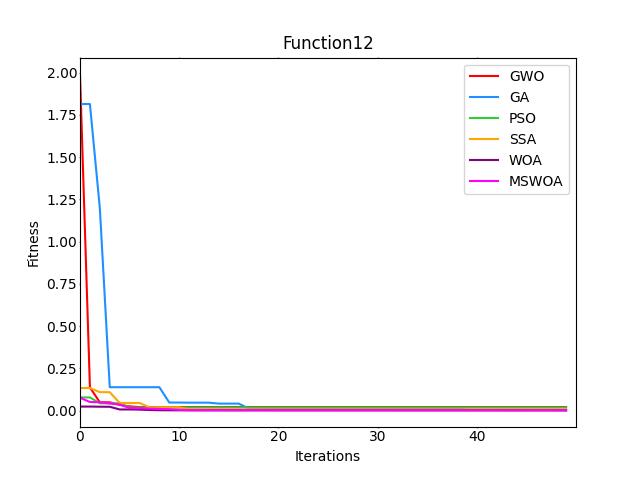

Supplement: Supplemental Information 1 [file peerj-cs-09-1729-s001.zip › code1/inteligent_algorithm_submit/Function12/func12/pic20.jpg]

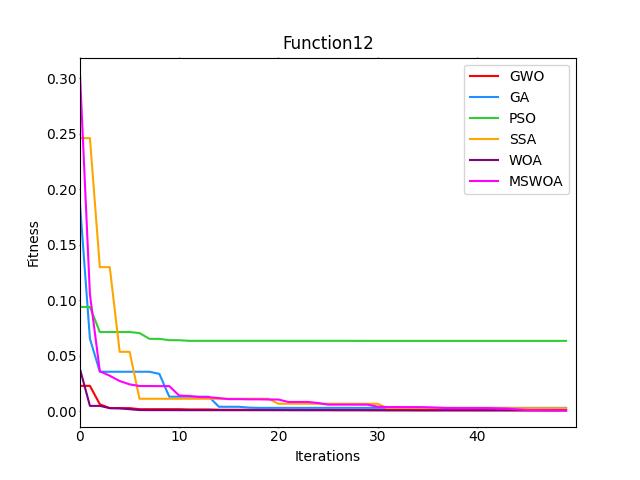

Supplement: Supplemental Information 1 [file peerj-cs-09-1729-s001.zip › code1/inteligent_algorithm_submit/Function12/func12/pic21.jpg]

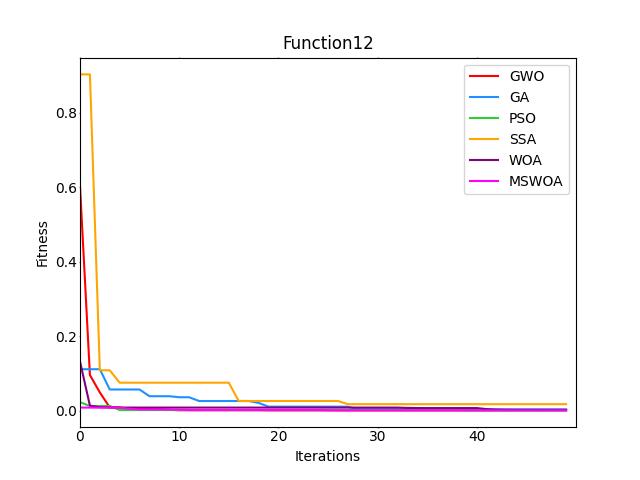

Supplement: Supplemental Information 1 [file peerj-cs-09-1729-s001.zip › code1/inteligent_algorithm_submit/Function12/func12/pic22.jpg]

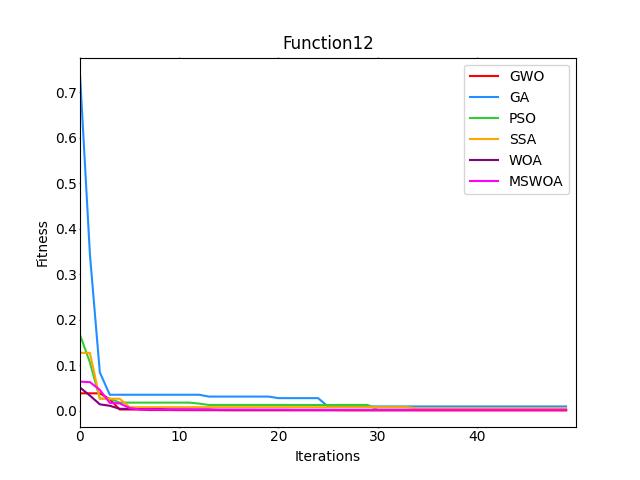

Supplement: Supplemental Information 1 [file peerj-cs-09-1729-s001.zip › code1/inteligent_algorithm_submit/Function12/func12/pic23.jpg]

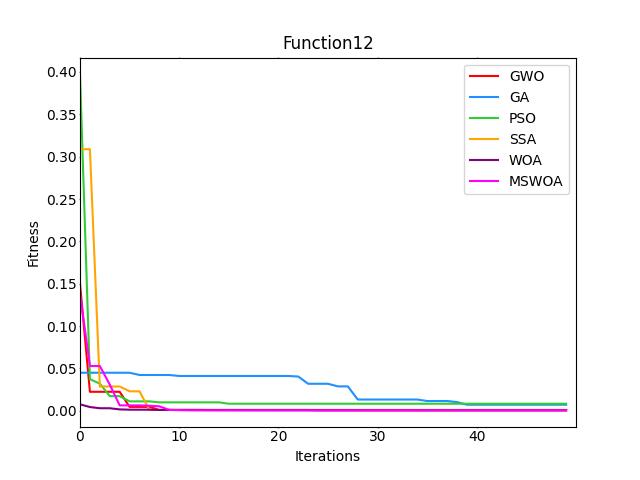

Supplement: Supplemental Information 1 [file peerj-cs-09-1729-s001.zip › code1/inteligent_algorithm_submit/Function12/func12/pic24.jpg]

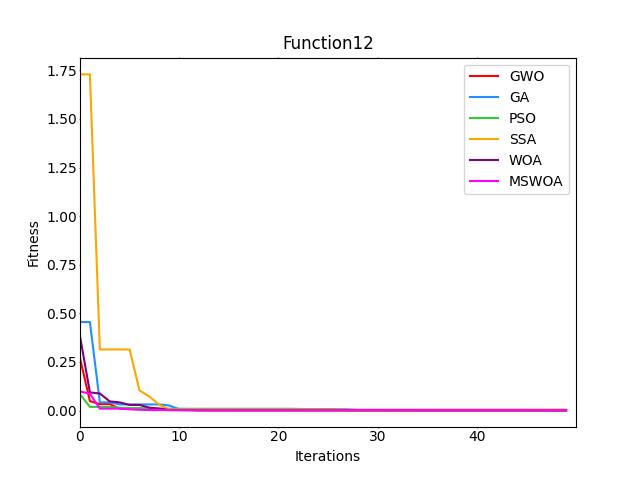

Supplement: Supplemental Information 1 [file peerj-cs-09-1729-s001.zip › code1/inteligent_algorithm_submit/Function12/func12/pic25.jpg]

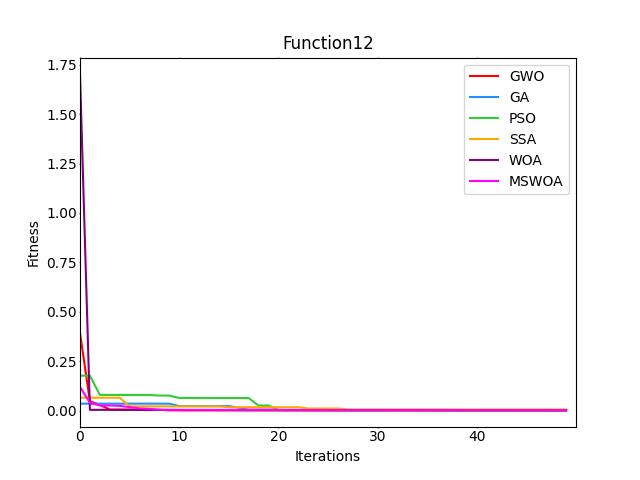

Supplement: Supplemental Information 1 [file peerj-cs-09-1729-s001.zip › code1/inteligent_algorithm_submit/Function12/func12/pic26.jpg]

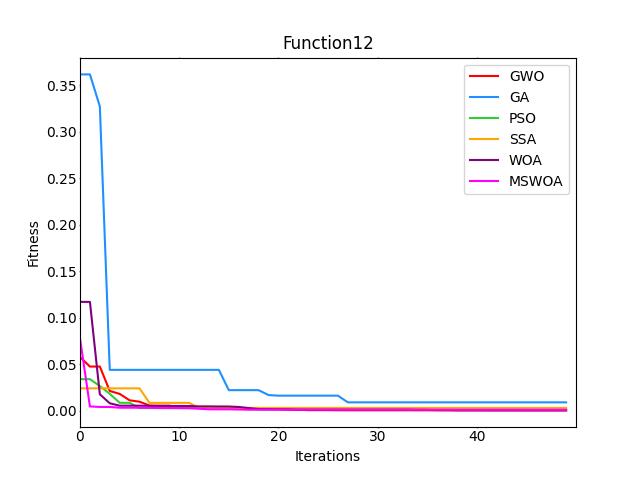

Supplement: Supplemental Information 1 [file peerj-cs-09-1729-s001.zip › code1/inteligent_algorithm_submit/Function12/func12/pic27.jpg]

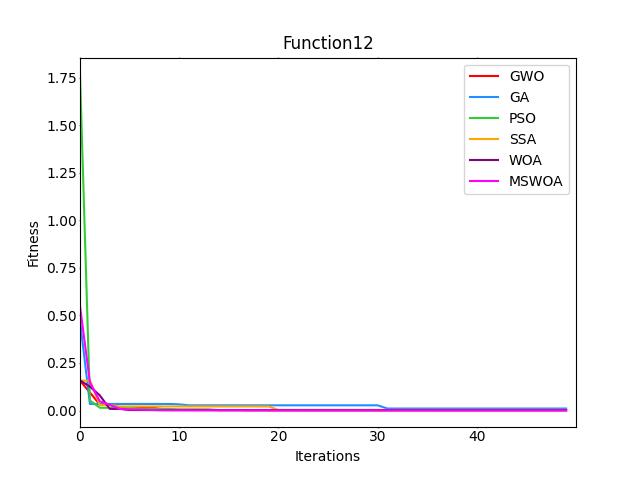

Supplement: Supplemental Information 1 [file peerj-cs-09-1729-s001.zip › code1/inteligent_algorithm_submit/Function12/func12/pic28.jpg]

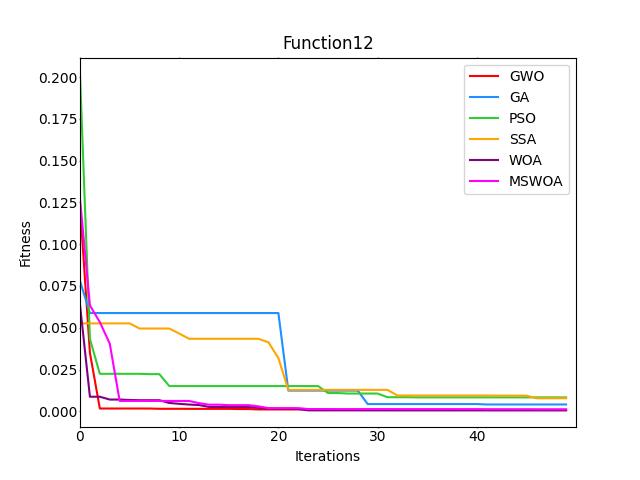

Supplement: Supplemental Information 1 [file peerj-cs-09-1729-s001.zip › code1/inteligent_algorithm_submit/Function12/func12/pic29.jpg]

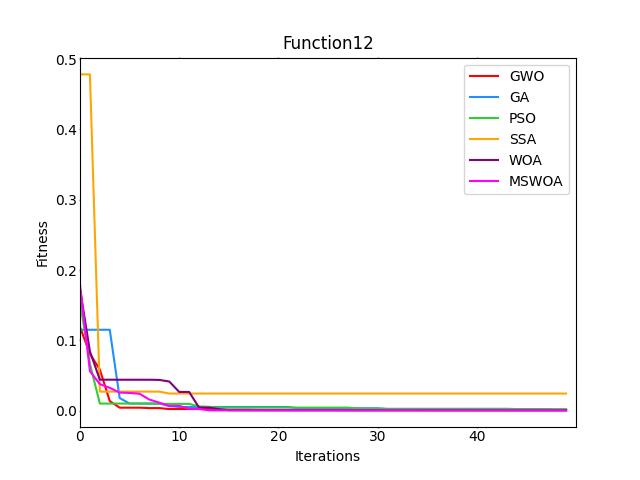

Supplement: Supplemental Information 1 [file peerj-cs-09-1729-s001.zip › code1/inteligent_algorithm_submit/Function12/func12/pic3.jpg]

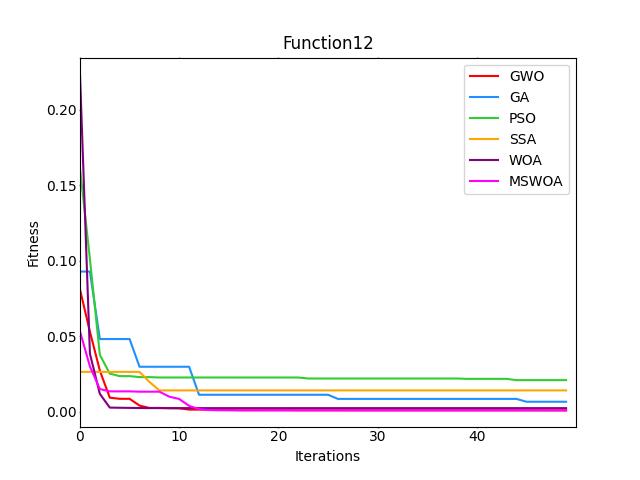

Supplement: Supplemental Information 1 [file peerj-cs-09-1729-s001.zip › code1/inteligent_algorithm_submit/Function12/func12/pic30.jpg]

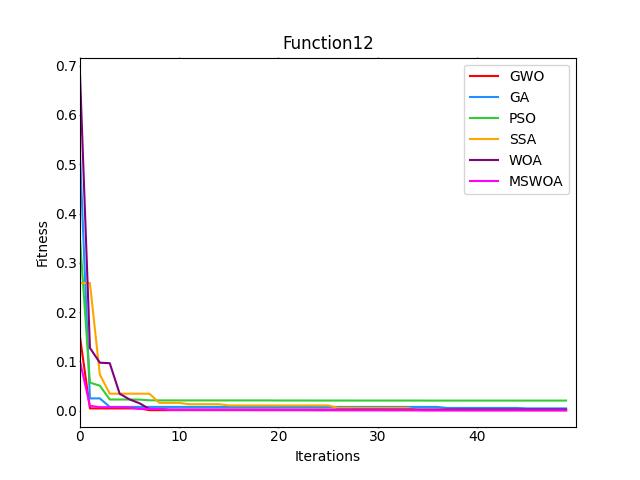

Supplement: Supplemental Information 1 [file peerj-cs-09-1729-s001.zip › code1/inteligent_algorithm_submit/Function12/func12/pic4.jpg]

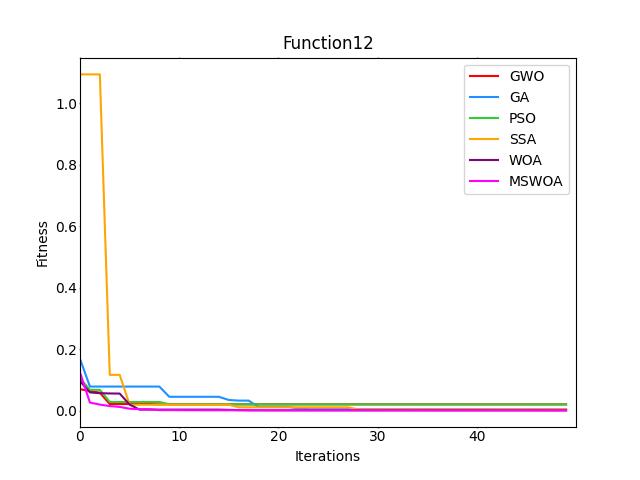

Supplement: Supplemental Information 1 [file peerj-cs-09-1729-s001.zip › code1/inteligent_algorithm_submit/Function12/func12/pic5.jpg]

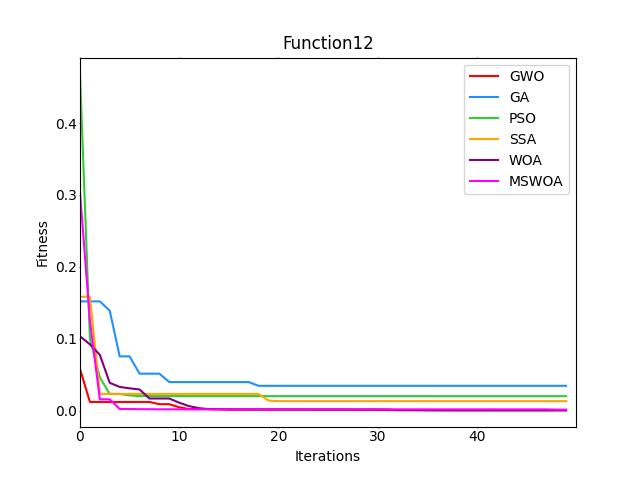

Supplement: Supplemental Information 1 [file peerj-cs-09-1729-s001.zip › code1/inteligent_algorithm_submit/Function12/func12/pic6.jpg]

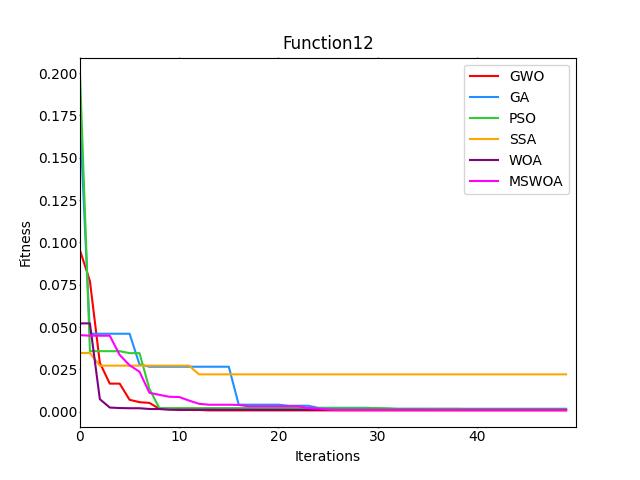

Supplement: Supplemental Information 1 [file peerj-cs-09-1729-s001.zip › code1/inteligent_algorithm_submit/Function12/func12/pic7.jpg]

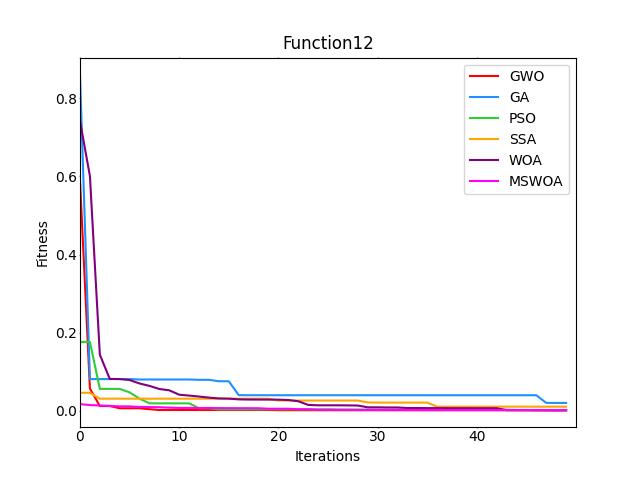

Supplement: Supplemental Information 1 [file peerj-cs-09-1729-s001.zip › code1/inteligent_algorithm_submit/Function12/func12/pic8.jpg]

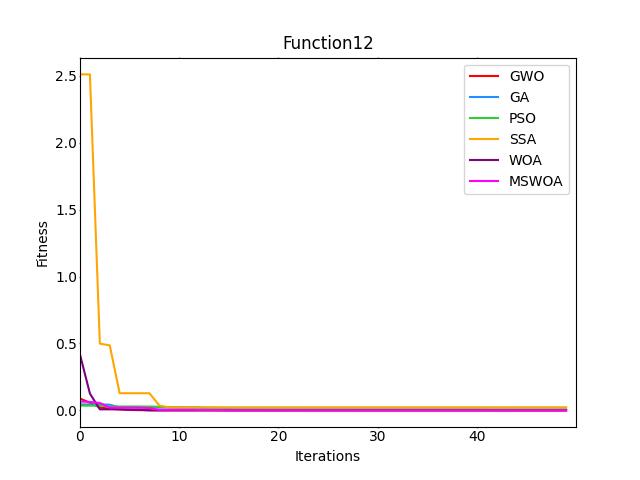

Supplement: Supplemental Information 1 [file peerj-cs-09-1729-s001.zip › code1/inteligent_algorithm_submit/Function12/func12/pic9.jpg]
