# Supplementary figures and images for: A novel network security situation assessment model based on multiple strategies whale optimization algorithm and bidirectional GRU (part 1 of 2)
Source: PeerJ Comput Sci. 2023 Dec 12;9:e1729. doi: 10.7717/peerj-cs.1729 (PMC10773833; doi:10.7717/peerj-cs.1729)

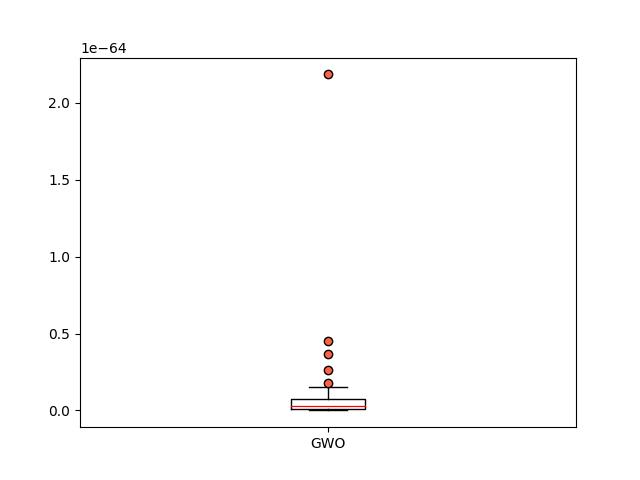

Supplement: Supplemental Information 1 [file peerj-cs-09-1729-s001.zip › code1/inteligent_algorithm_submit/Function1/boxplot_func1/pic1.jpg]

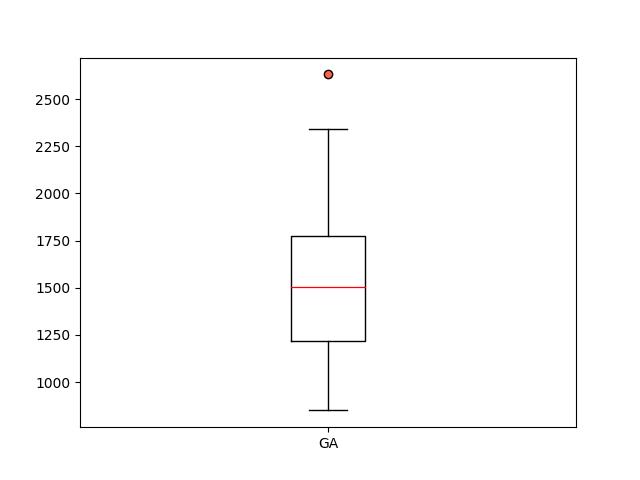

Supplement: Supplemental Information 1 [file peerj-cs-09-1729-s001.zip › code1/inteligent_algorithm_submit/Function1/boxplot_func1/pic2.jpg]

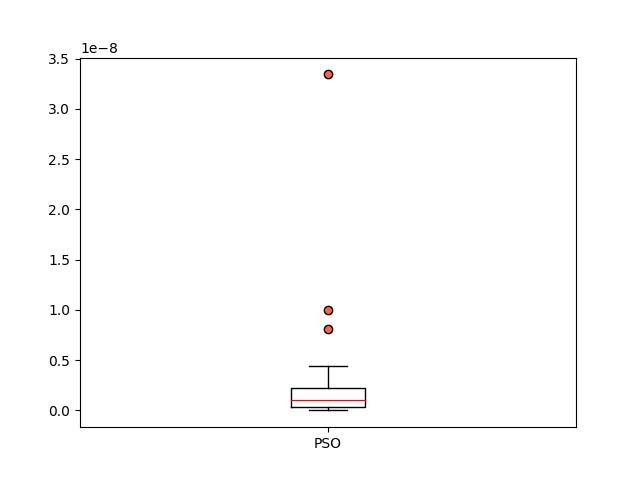

Supplement: Supplemental Information 1 [file peerj-cs-09-1729-s001.zip › code1/inteligent_algorithm_submit/Function1/boxplot_func1/pic3.jpg]

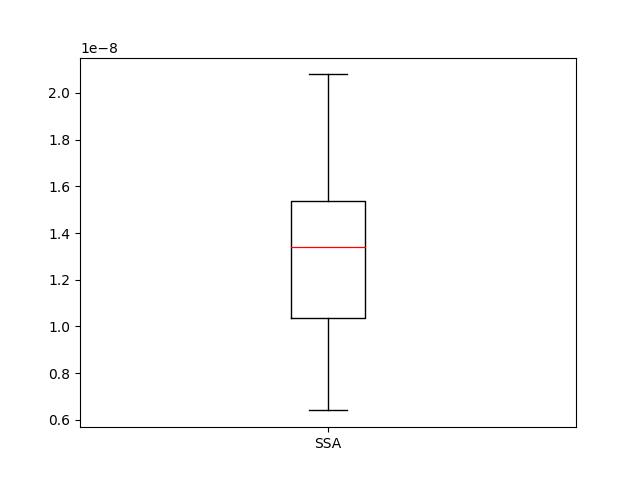

Supplement: Supplemental Information 1 [file peerj-cs-09-1729-s001.zip › code1/inteligent_algorithm_submit/Function1/boxplot_func1/pic4.jpg]

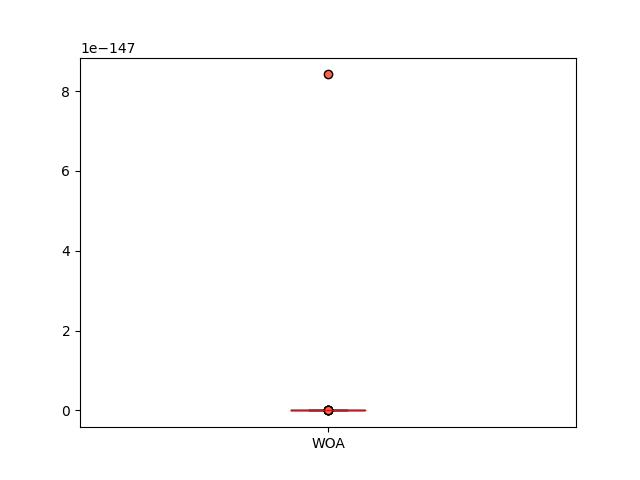

Supplement: Supplemental Information 1 [file peerj-cs-09-1729-s001.zip › code1/inteligent_algorithm_submit/Function1/boxplot_func1/pic5.jpg]

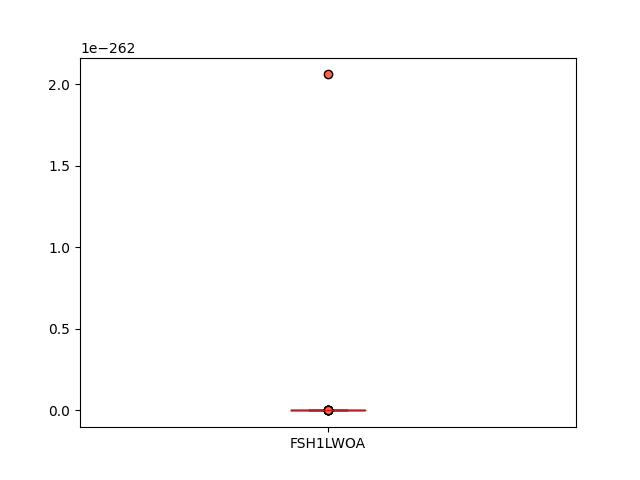

Supplement: Supplemental Information 1 [file peerj-cs-09-1729-s001.zip › code1/inteligent_algorithm_submit/Function1/boxplot_func1/pic6.jpg]

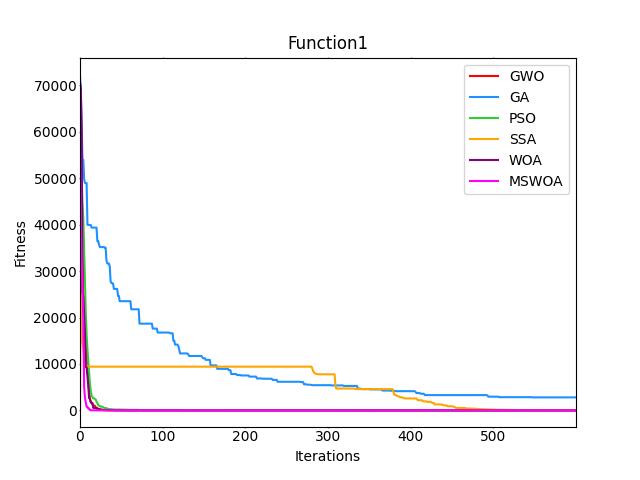

Supplement: Supplemental Information 1 [file peerj-cs-09-1729-s001.zip › code1/inteligent_algorithm_submit/Function1/func1/pic1.jpg]

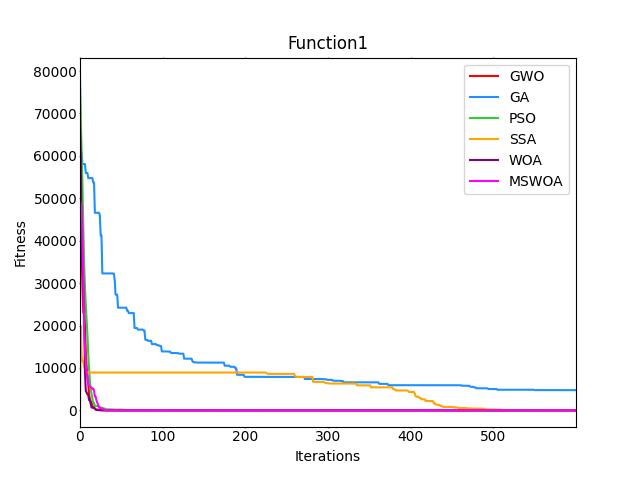

Supplement: Supplemental Information 1 [file peerj-cs-09-1729-s001.zip › code1/inteligent_algorithm_submit/Function1/func1/pic10.jpg]

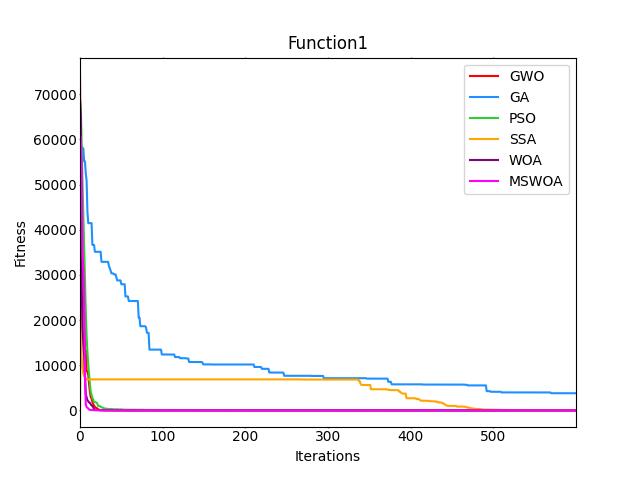

Supplement: Supplemental Information 1 [file peerj-cs-09-1729-s001.zip › code1/inteligent_algorithm_submit/Function1/func1/pic11.jpg]

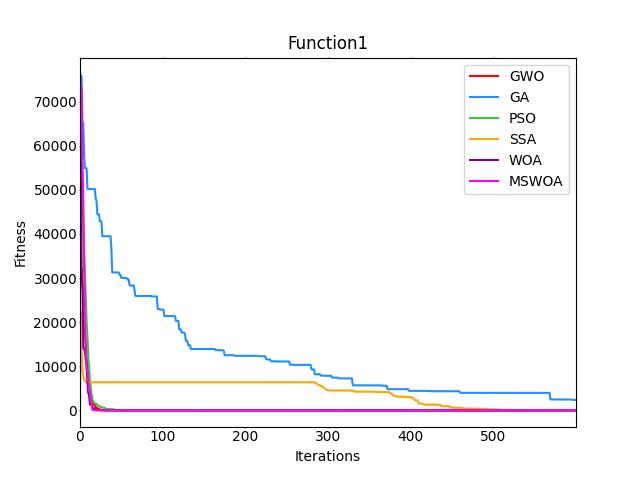

Supplement: Supplemental Information 1 [file peerj-cs-09-1729-s001.zip › code1/inteligent_algorithm_submit/Function1/func1/pic12.jpg]

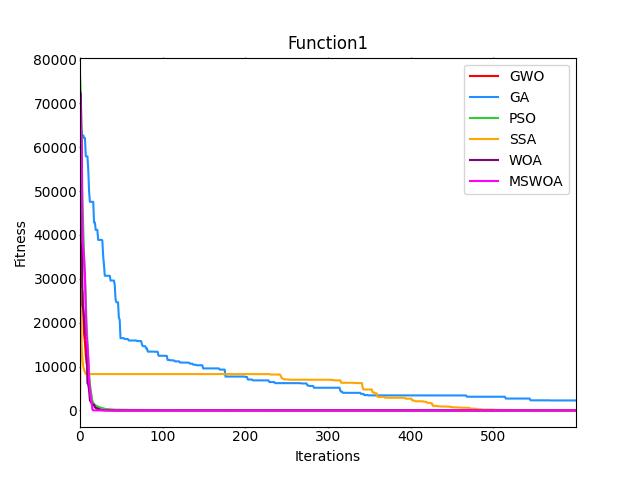

Supplement: Supplemental Information 1 [file peerj-cs-09-1729-s001.zip › code1/inteligent_algorithm_submit/Function1/func1/pic13.jpg]

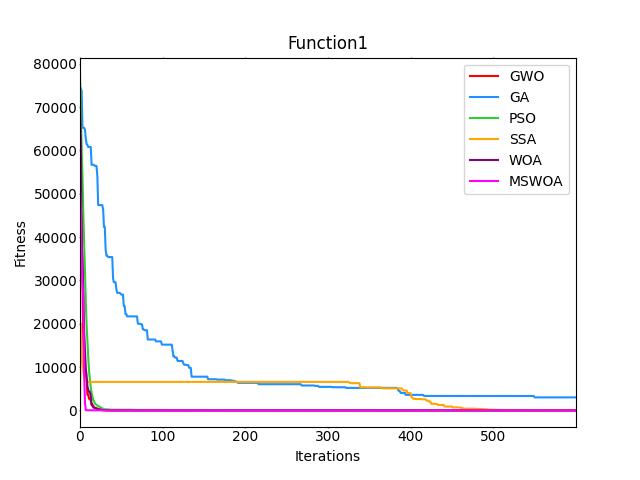

Supplement: Supplemental Information 1 [file peerj-cs-09-1729-s001.zip › code1/inteligent_algorithm_submit/Function1/func1/pic14.jpg]

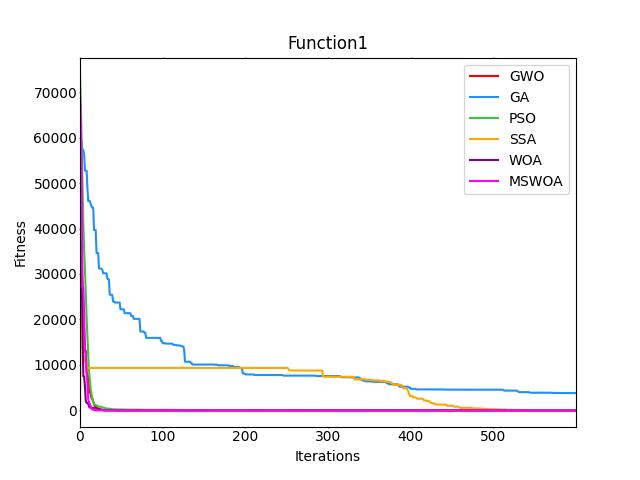

Supplement: Supplemental Information 1 [file peerj-cs-09-1729-s001.zip › code1/inteligent_algorithm_submit/Function1/func1/pic15.jpg]

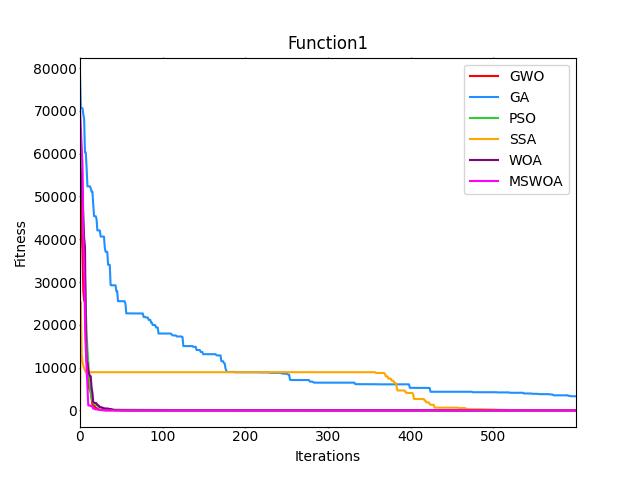

Supplement: Supplemental Information 1 [file peerj-cs-09-1729-s001.zip › code1/inteligent_algorithm_submit/Function1/func1/pic16.jpg]

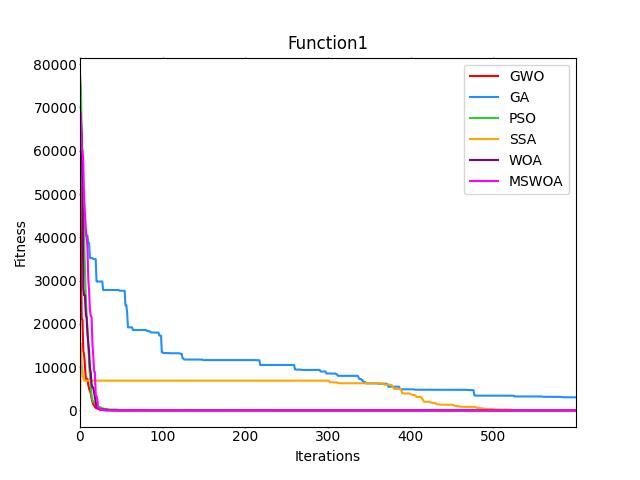

Supplement: Supplemental Information 1 [file peerj-cs-09-1729-s001.zip › code1/inteligent_algorithm_submit/Function1/func1/pic17.jpg]

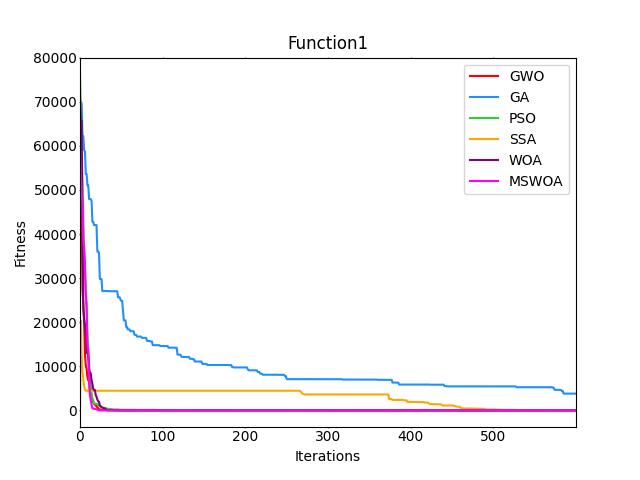

Supplement: Supplemental Information 1 [file peerj-cs-09-1729-s001.zip › code1/inteligent_algorithm_submit/Function1/func1/pic18.jpg]

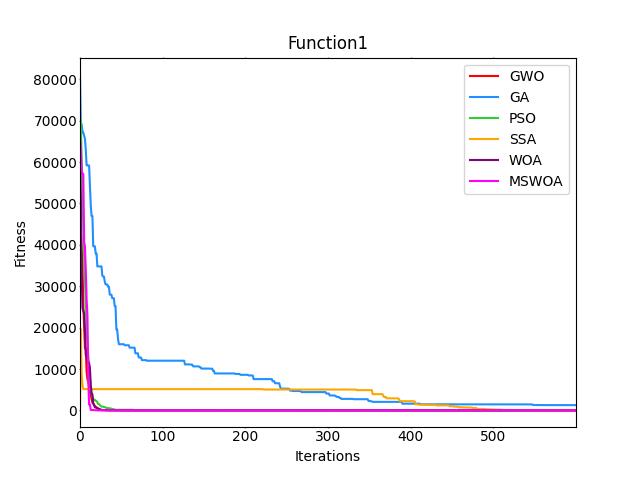

Supplement: Supplemental Information 1 [file peerj-cs-09-1729-s001.zip › code1/inteligent_algorithm_submit/Function1/func1/pic19.jpg]

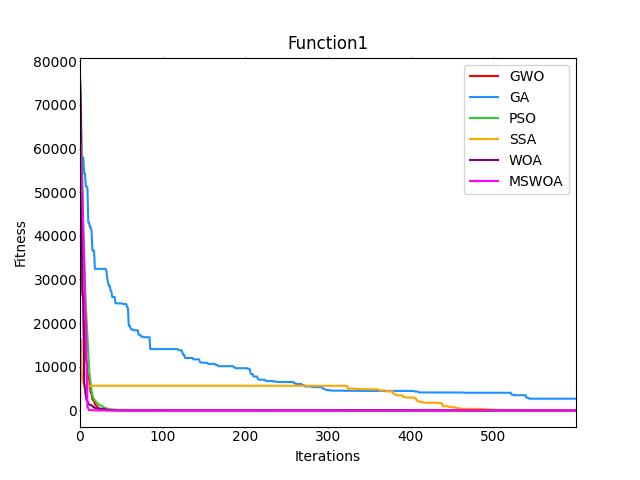

Supplement: Supplemental Information 1 [file peerj-cs-09-1729-s001.zip › code1/inteligent_algorithm_submit/Function1/func1/pic2.jpg]

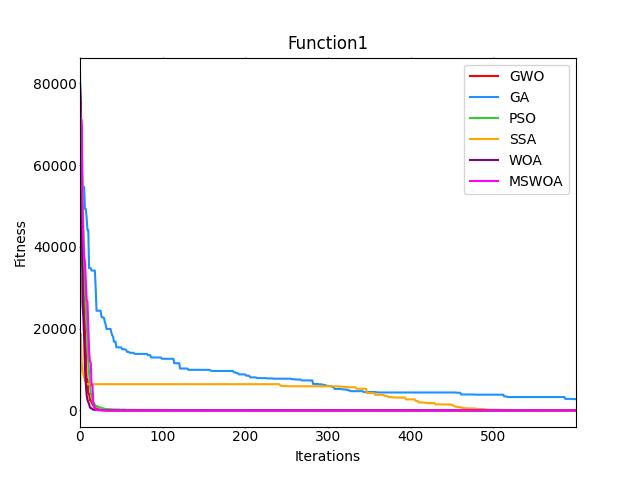

Supplement: Supplemental Information 1 [file peerj-cs-09-1729-s001.zip › code1/inteligent_algorithm_submit/Function1/func1/pic20.jpg]

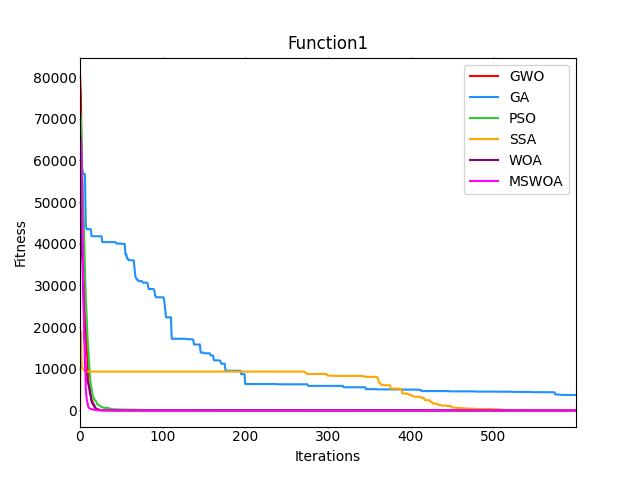

Supplement: Supplemental Information 1 [file peerj-cs-09-1729-s001.zip › code1/inteligent_algorithm_submit/Function1/func1/pic21.jpg]

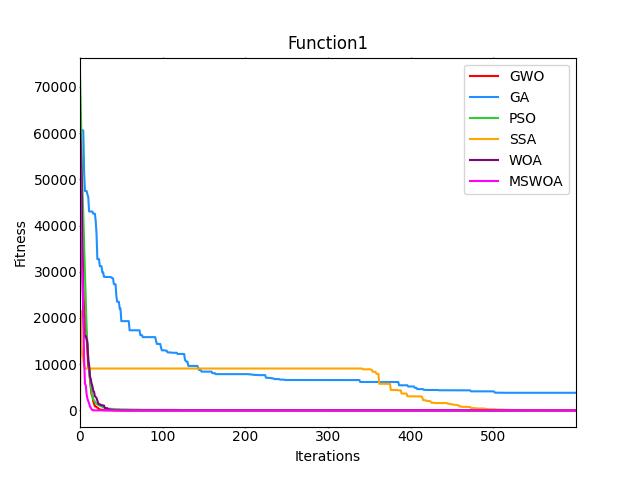

Supplement: Supplemental Information 1 [file peerj-cs-09-1729-s001.zip › code1/inteligent_algorithm_submit/Function1/func1/pic22.jpg]

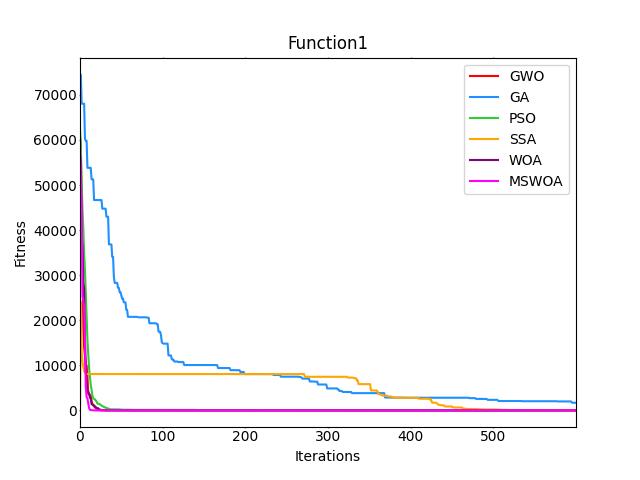

Supplement: Supplemental Information 1 [file peerj-cs-09-1729-s001.zip › code1/inteligent_algorithm_submit/Function1/func1/pic23.jpg]

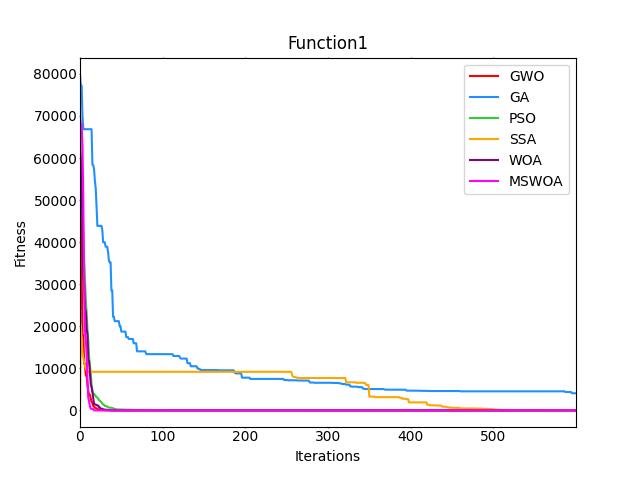

Supplement: Supplemental Information 1 [file peerj-cs-09-1729-s001.zip › code1/inteligent_algorithm_submit/Function1/func1/pic24.jpg]

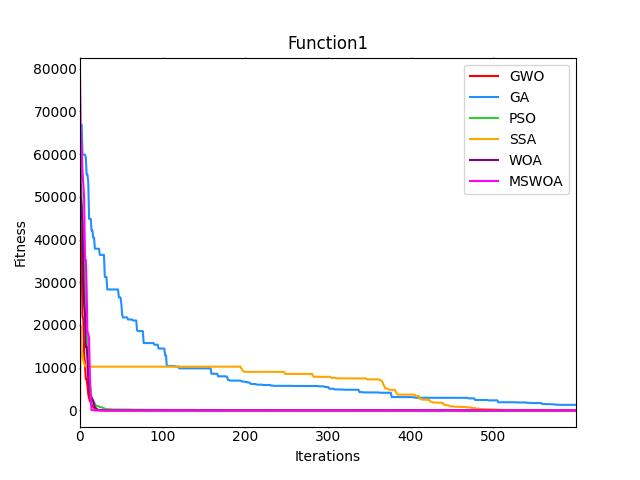

Supplement: Supplemental Information 1 [file peerj-cs-09-1729-s001.zip › code1/inteligent_algorithm_submit/Function1/func1/pic25.jpg]

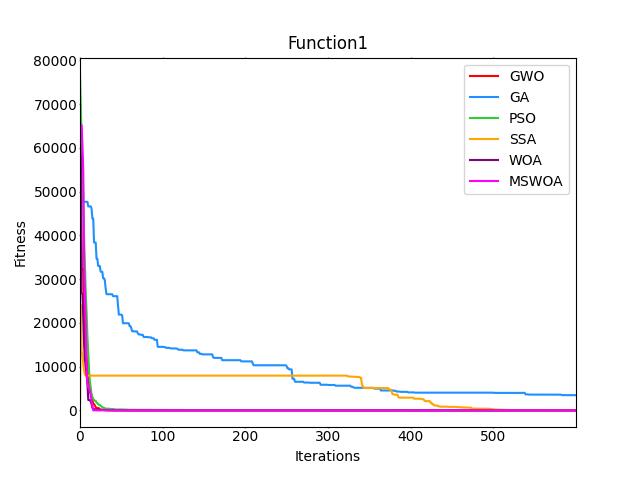

Supplement: Supplemental Information 1 [file peerj-cs-09-1729-s001.zip › code1/inteligent_algorithm_submit/Function1/func1/pic26.jpg]

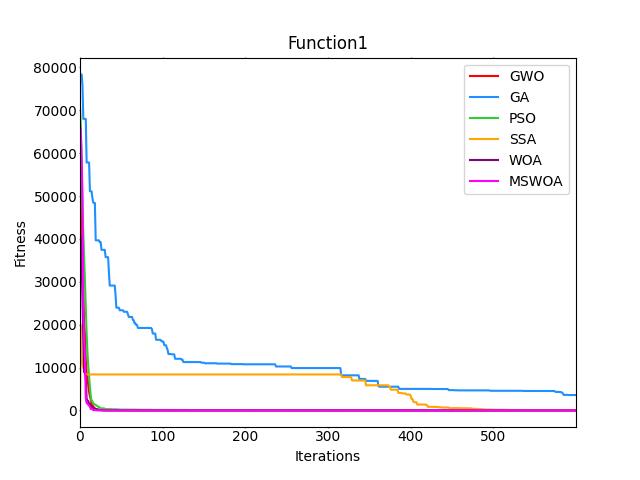

Supplement: Supplemental Information 1 [file peerj-cs-09-1729-s001.zip › code1/inteligent_algorithm_submit/Function1/func1/pic27.jpg]

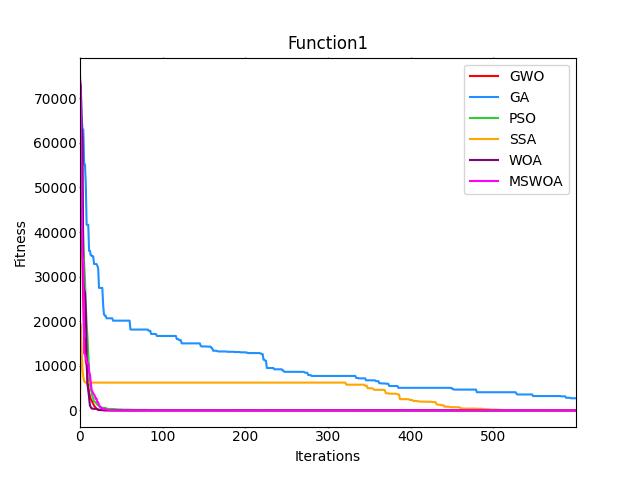

Supplement: Supplemental Information 1 [file peerj-cs-09-1729-s001.zip › code1/inteligent_algorithm_submit/Function1/func1/pic28.jpg]

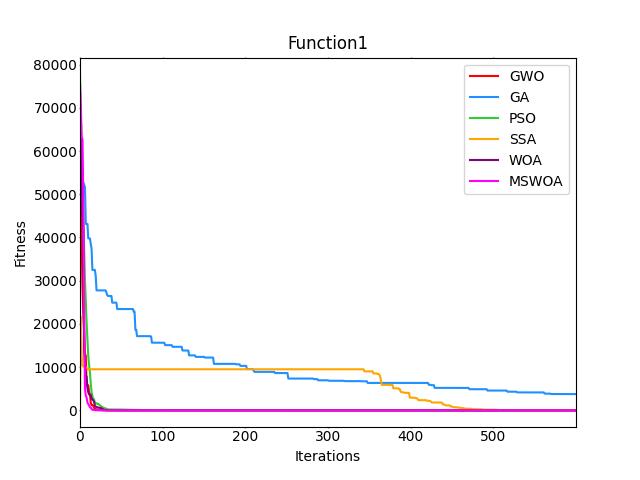

Supplement: Supplemental Information 1 [file peerj-cs-09-1729-s001.zip › code1/inteligent_algorithm_submit/Function1/func1/pic29.jpg]

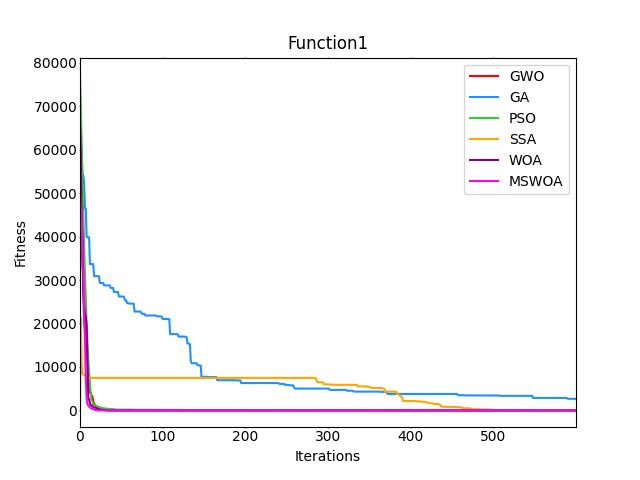

Supplement: Supplemental Information 1 [file peerj-cs-09-1729-s001.zip › code1/inteligent_algorithm_submit/Function1/func1/pic3.jpg]

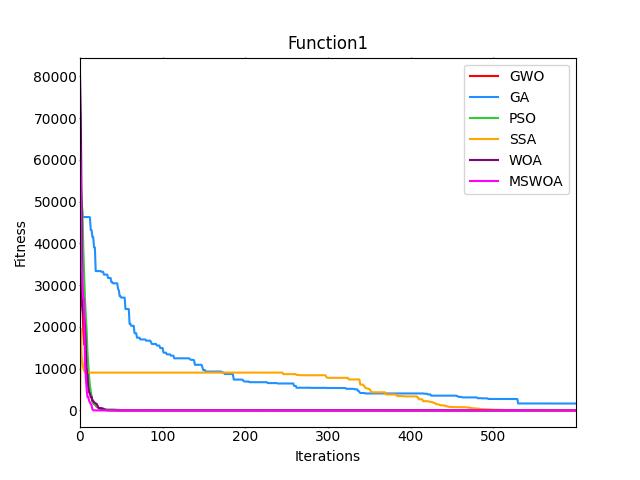

Supplement: Supplemental Information 1 [file peerj-cs-09-1729-s001.zip › code1/inteligent_algorithm_submit/Function1/func1/pic30.jpg]

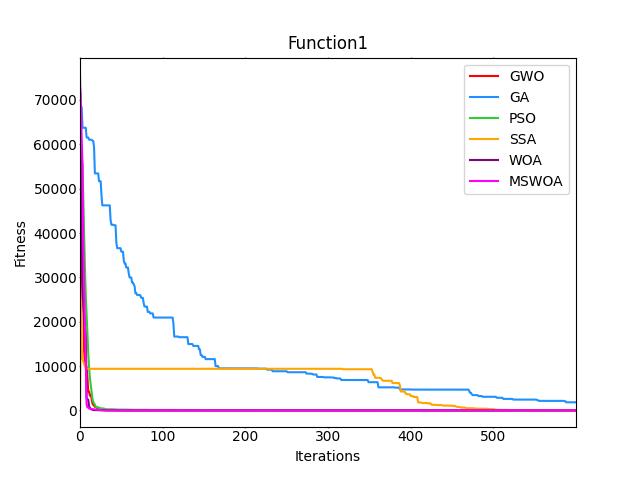

Supplement: Supplemental Information 1 [file peerj-cs-09-1729-s001.zip › code1/inteligent_algorithm_submit/Function1/func1/pic4.jpg]

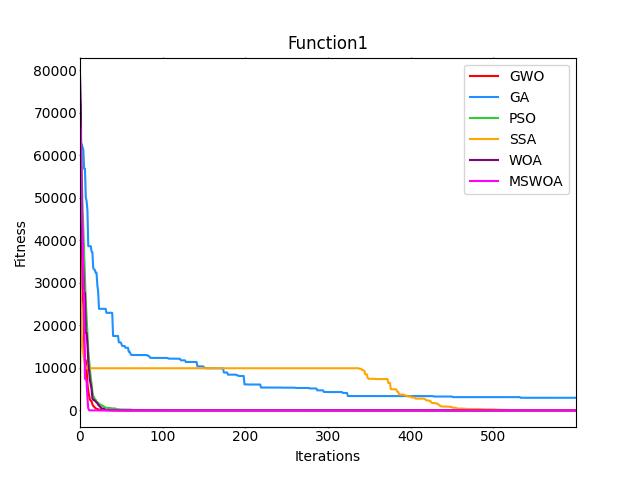

Supplement: Supplemental Information 1 [file peerj-cs-09-1729-s001.zip › code1/inteligent_algorithm_submit/Function1/func1/pic5.jpg]

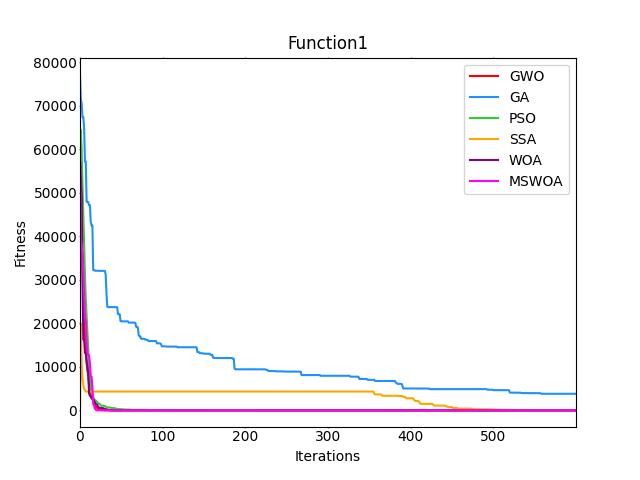

Supplement: Supplemental Information 1 [file peerj-cs-09-1729-s001.zip › code1/inteligent_algorithm_submit/Function1/func1/pic6.jpg]

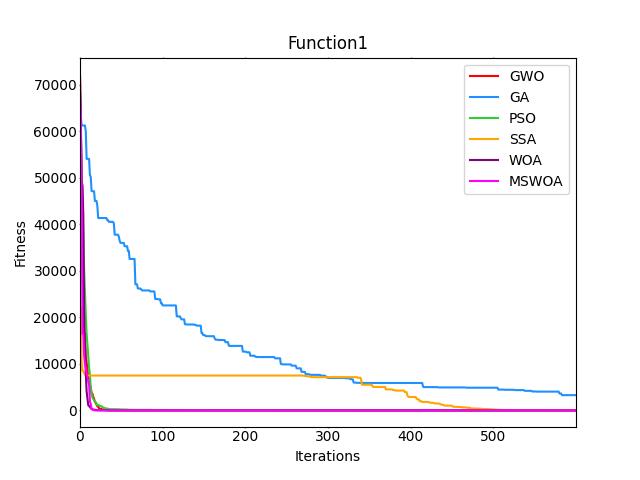

Supplement: Supplemental Information 1 [file peerj-cs-09-1729-s001.zip › code1/inteligent_algorithm_submit/Function1/func1/pic7.jpg]

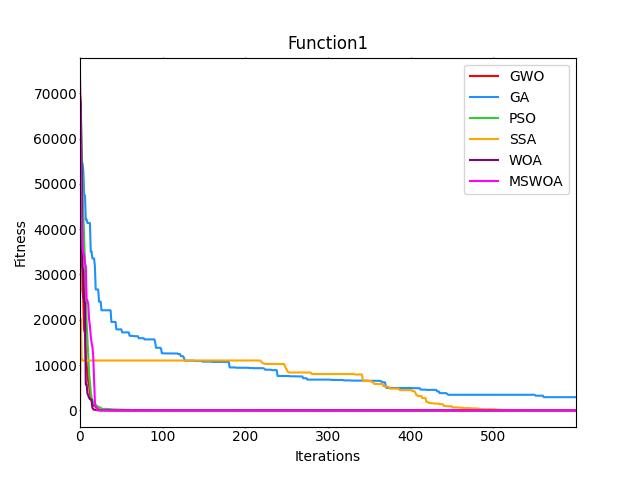

Supplement: Supplemental Information 1 [file peerj-cs-09-1729-s001.zip › code1/inteligent_algorithm_submit/Function1/func1/pic8.jpg]

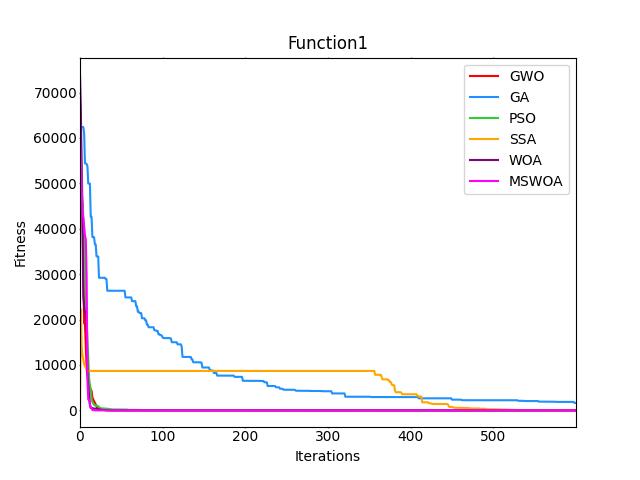

Supplement: Supplemental Information 1 [file peerj-cs-09-1729-s001.zip › code1/inteligent_algorithm_submit/Function1/func1/pic9.jpg]

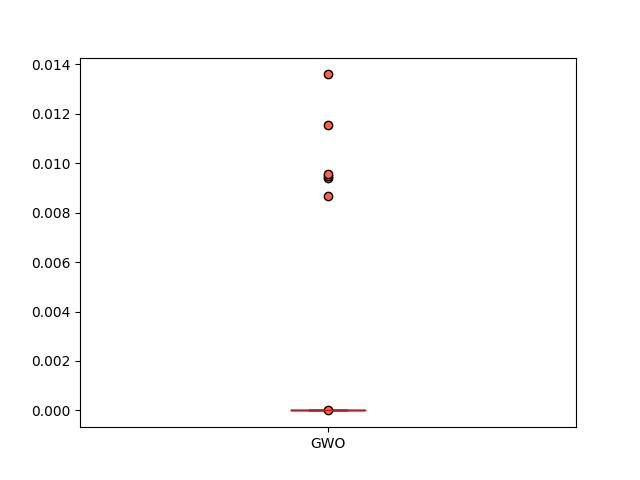

Supplement: Supplemental Information 1 [file peerj-cs-09-1729-s001.zip › code1/inteligent_algorithm_submit/Function10/boxplot_func10/pic1.jpg]

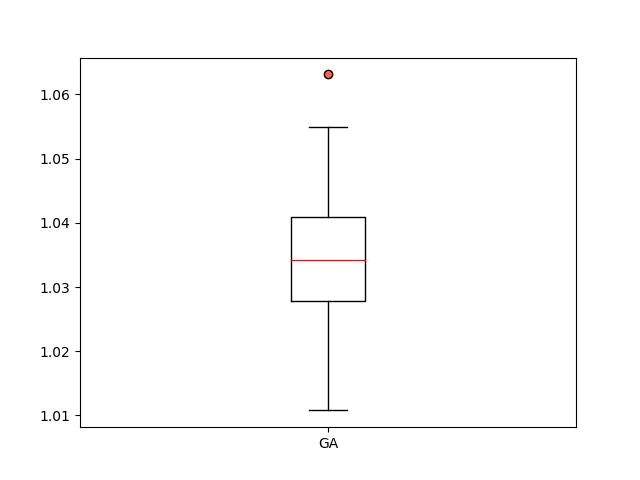

Supplement: Supplemental Information 1 [file peerj-cs-09-1729-s001.zip › code1/inteligent_algorithm_submit/Function10/boxplot_func10/pic2.jpg]

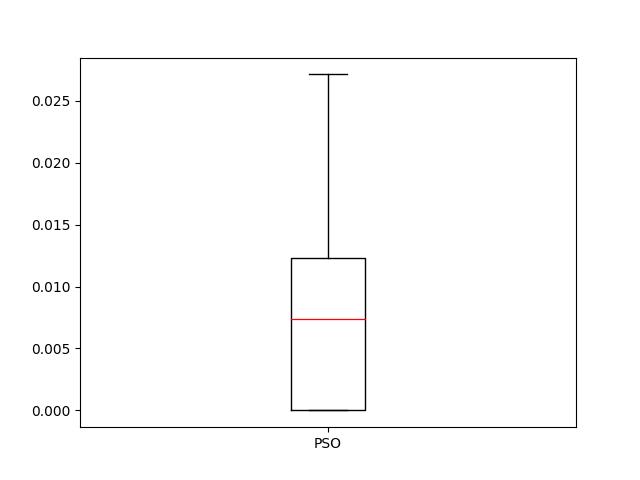

Supplement: Supplemental Information 1 [file peerj-cs-09-1729-s001.zip › code1/inteligent_algorithm_submit/Function10/boxplot_func10/pic3.jpg]

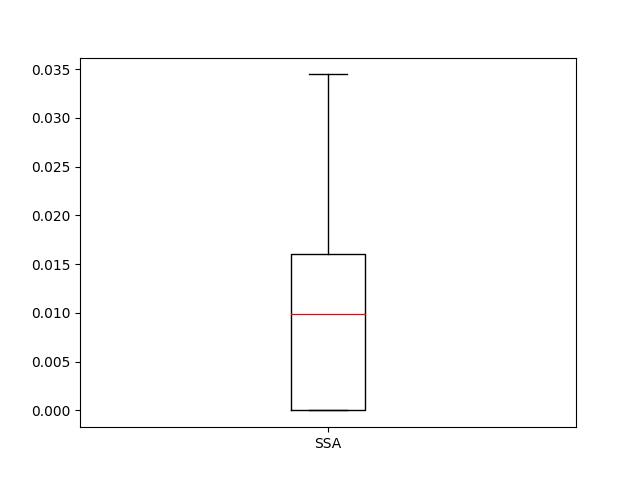

Supplement: Supplemental Information 1 [file peerj-cs-09-1729-s001.zip › code1/inteligent_algorithm_submit/Function10/boxplot_func10/pic4.jpg]

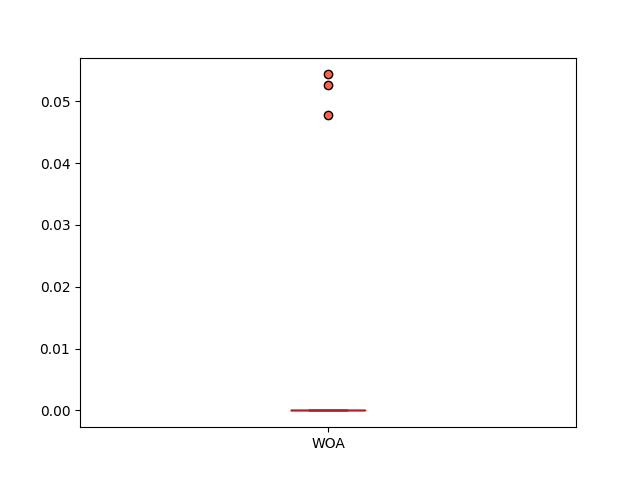

Supplement: Supplemental Information 1 [file peerj-cs-09-1729-s001.zip › code1/inteligent_algorithm_submit/Function10/boxplot_func10/pic5.jpg]

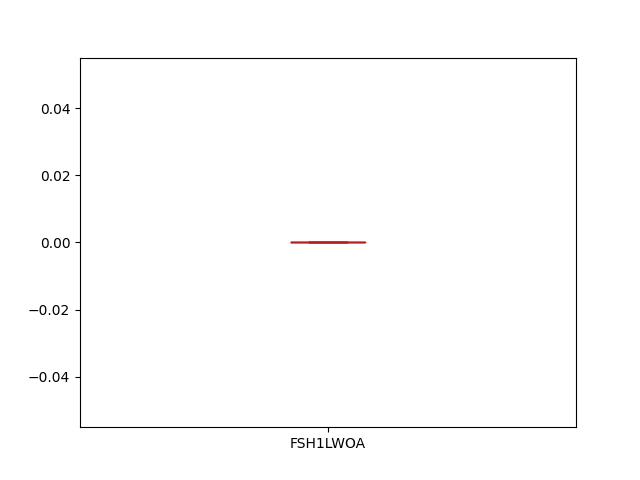

Supplement: Supplemental Information 1 [file peerj-cs-09-1729-s001.zip › code1/inteligent_algorithm_submit/Function10/boxplot_func10/pic6.jpg]

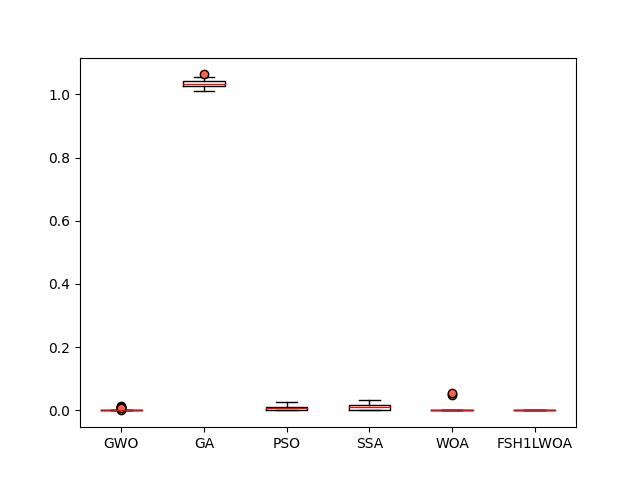

Supplement: Supplemental Information 1 [file peerj-cs-09-1729-s001.zip › code1/inteligent_algorithm_submit/Function10/boxplot_func10/pic_total.jpg]

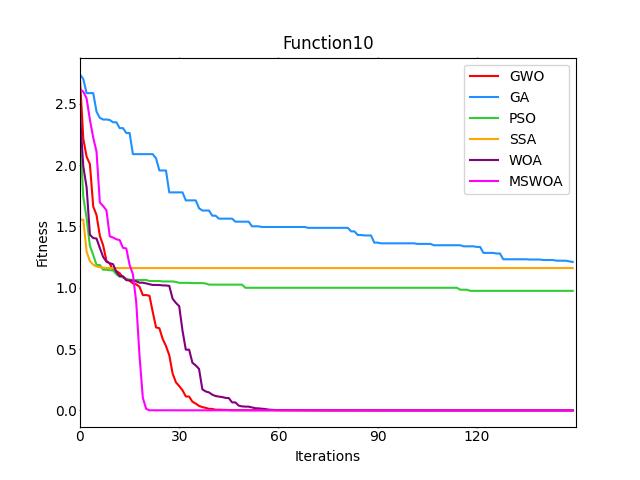

Supplement: Supplemental Information 1 [file peerj-cs-09-1729-s001.zip › code1/inteligent_algorithm_submit/Function10/func10/pic1.jpg]

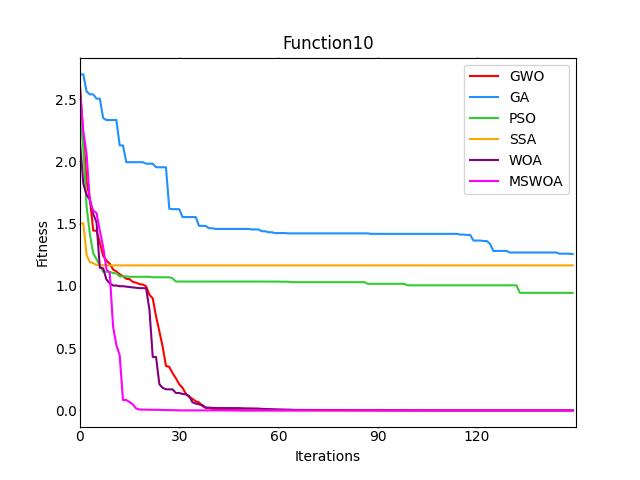

Supplement: Supplemental Information 1 [file peerj-cs-09-1729-s001.zip › code1/inteligent_algorithm_submit/Function10/func10/pic10.jpg]

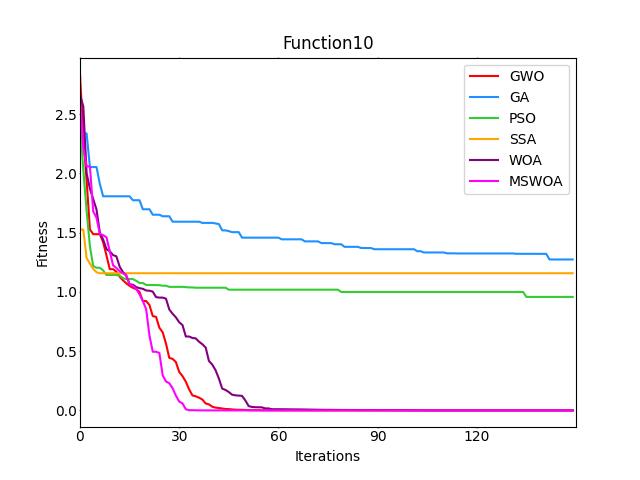

Supplement: Supplemental Information 1 [file peerj-cs-09-1729-s001.zip › code1/inteligent_algorithm_submit/Function10/func10/pic11.jpg]

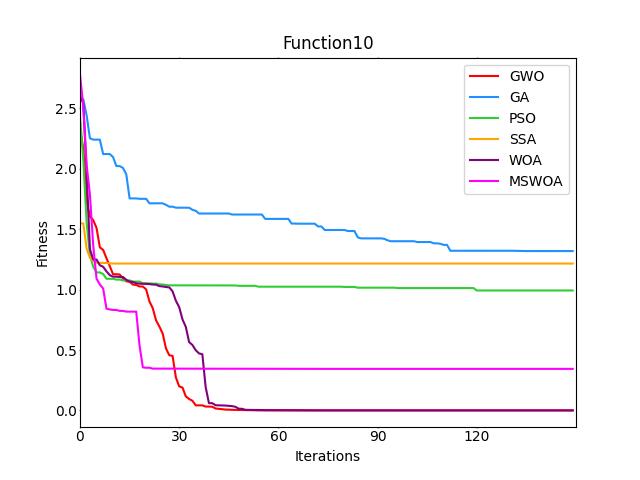

Supplement: Supplemental Information 1 [file peerj-cs-09-1729-s001.zip › code1/inteligent_algorithm_submit/Function10/func10/pic12.jpg]

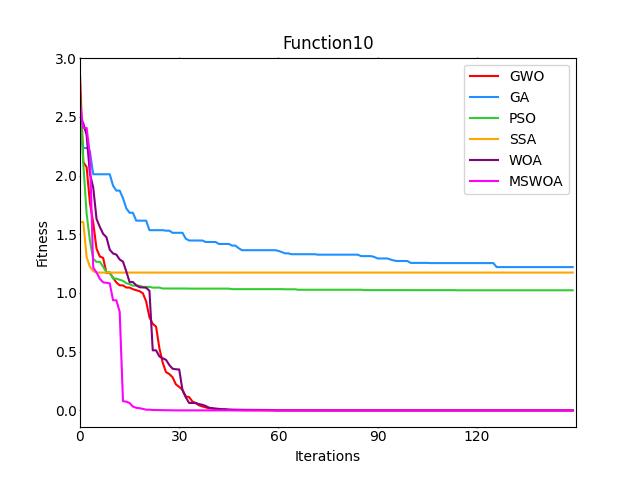

Supplement: Supplemental Information 1 [file peerj-cs-09-1729-s001.zip › code1/inteligent_algorithm_submit/Function10/func10/pic13.jpg]

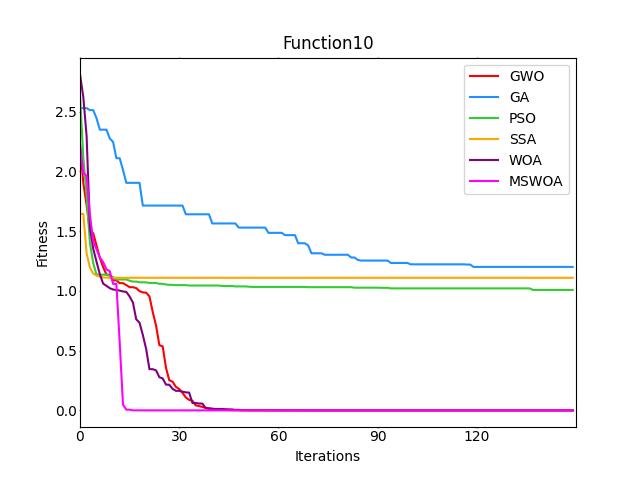

Supplement: Supplemental Information 1 [file peerj-cs-09-1729-s001.zip › code1/inteligent_algorithm_submit/Function10/func10/pic14.jpg]

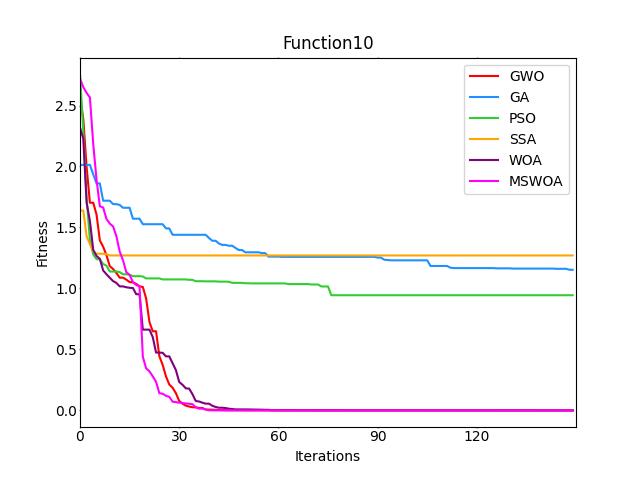

Supplement: Supplemental Information 1 [file peerj-cs-09-1729-s001.zip › code1/inteligent_algorithm_submit/Function10/func10/pic15.jpg]

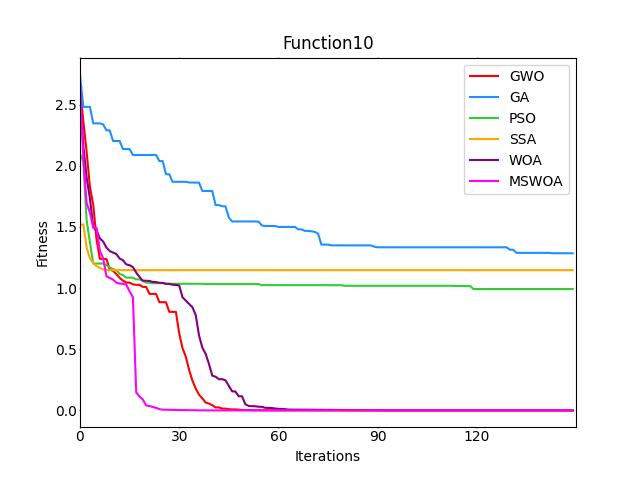

Supplement: Supplemental Information 1 [file peerj-cs-09-1729-s001.zip › code1/inteligent_algorithm_submit/Function10/func10/pic16.jpg]

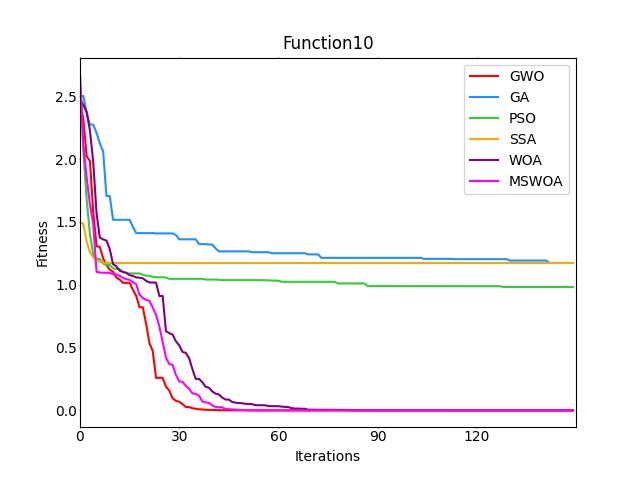

Supplement: Supplemental Information 1 [file peerj-cs-09-1729-s001.zip › code1/inteligent_algorithm_submit/Function10/func10/pic17.jpg]

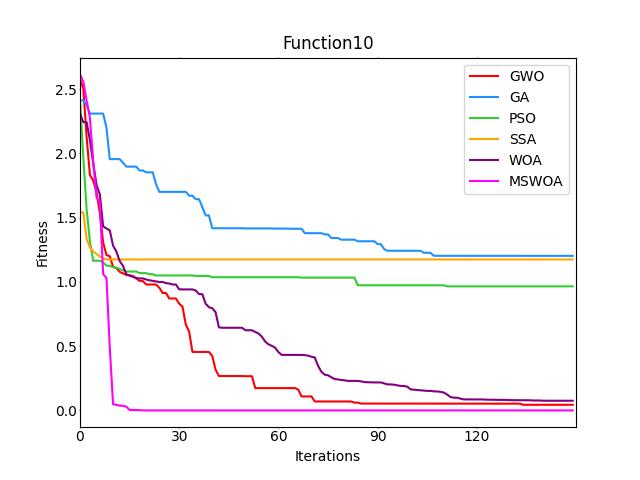

Supplement: Supplemental Information 1 [file peerj-cs-09-1729-s001.zip › code1/inteligent_algorithm_submit/Function10/func10/pic18.jpg]

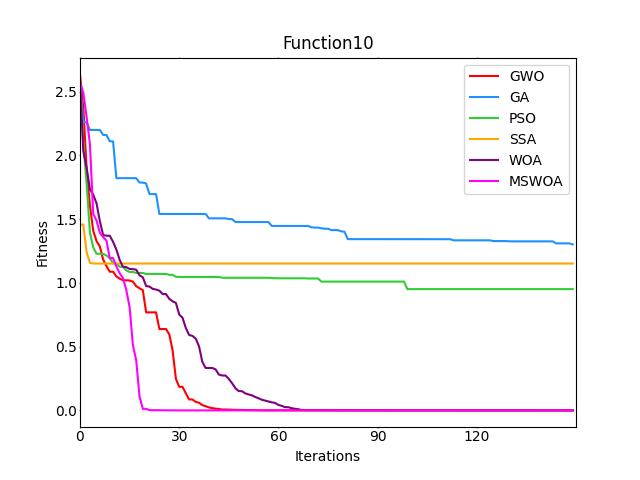

Supplement: Supplemental Information 1 [file peerj-cs-09-1729-s001.zip › code1/inteligent_algorithm_submit/Function10/func10/pic19.jpg]

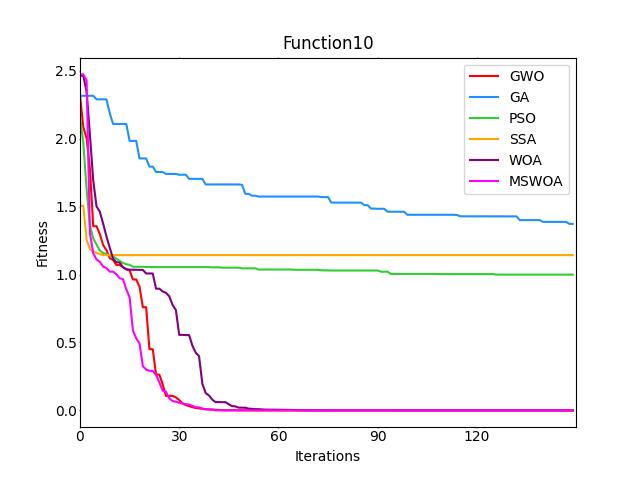

Supplement: Supplemental Information 1 [file peerj-cs-09-1729-s001.zip › code1/inteligent_algorithm_submit/Function10/func10/pic2.jpg]

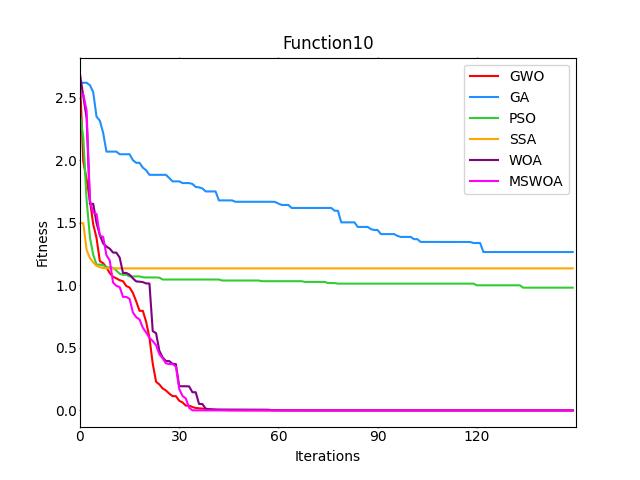

Supplement: Supplemental Information 1 [file peerj-cs-09-1729-s001.zip › code1/inteligent_algorithm_submit/Function10/func10/pic20.jpg]

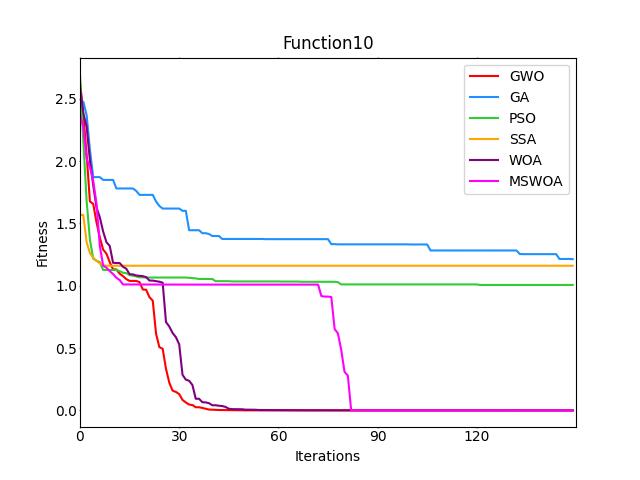

Supplement: Supplemental Information 1 [file peerj-cs-09-1729-s001.zip › code1/inteligent_algorithm_submit/Function10/func10/pic21.jpg]

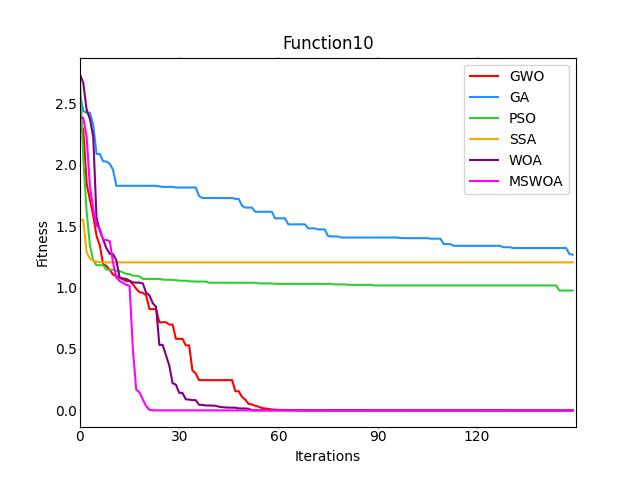

Supplement: Supplemental Information 1 [file peerj-cs-09-1729-s001.zip › code1/inteligent_algorithm_submit/Function10/func10/pic22.jpg]

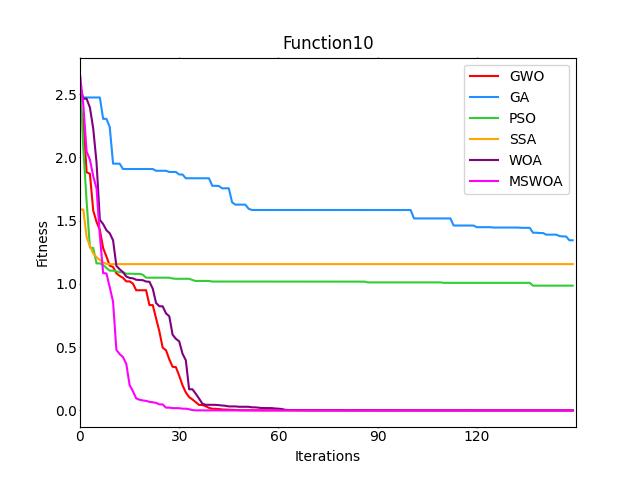

Supplement: Supplemental Information 1 [file peerj-cs-09-1729-s001.zip › code1/inteligent_algorithm_submit/Function10/func10/pic23.jpg]

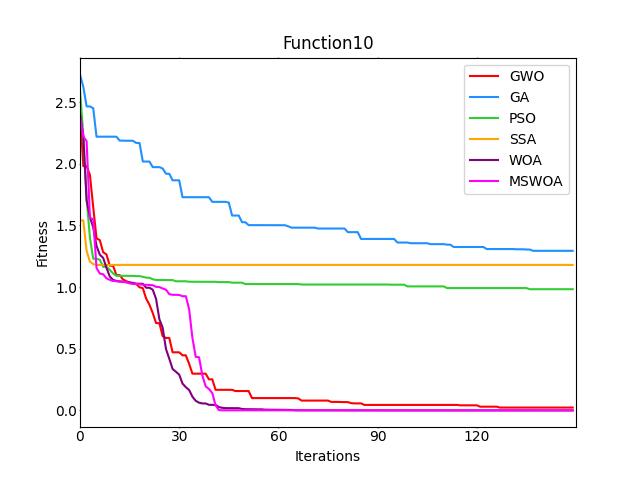

Supplement: Supplemental Information 1 [file peerj-cs-09-1729-s001.zip › code1/inteligent_algorithm_submit/Function10/func10/pic24.jpg]

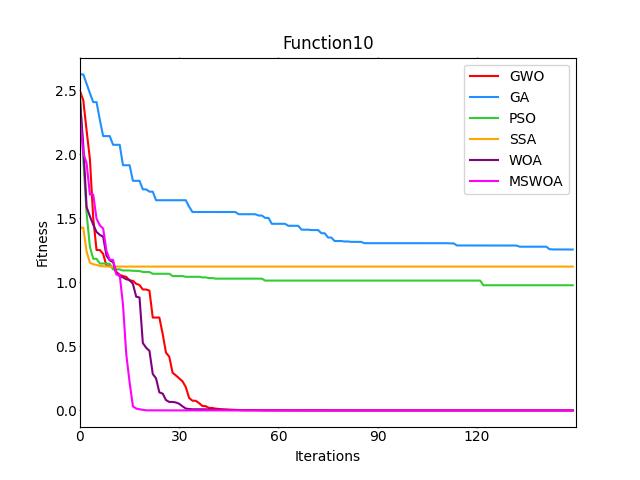

Supplement: Supplemental Information 1 [file peerj-cs-09-1729-s001.zip › code1/inteligent_algorithm_submit/Function10/func10/pic25.jpg]

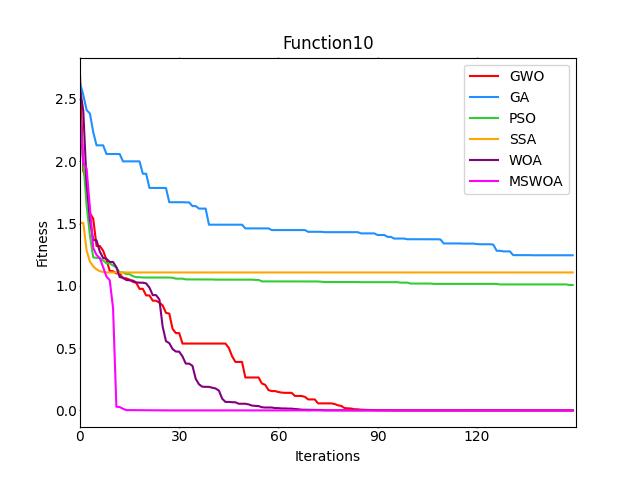

Supplement: Supplemental Information 1 [file peerj-cs-09-1729-s001.zip › code1/inteligent_algorithm_submit/Function10/func10/pic26.jpg]

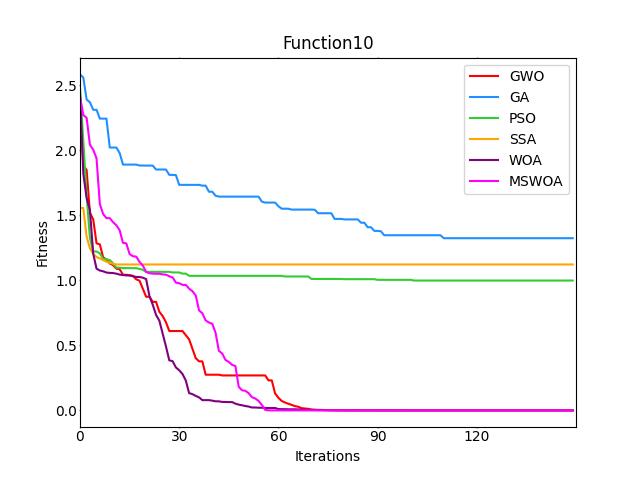

Supplement: Supplemental Information 1 [file peerj-cs-09-1729-s001.zip › code1/inteligent_algorithm_submit/Function10/func10/pic27.jpg]

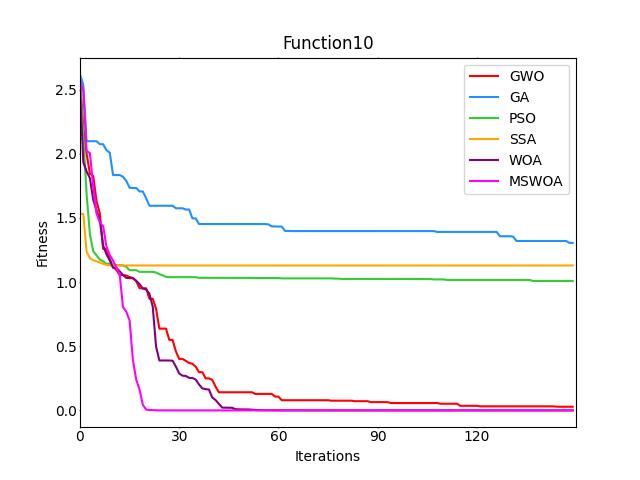

Supplement: Supplemental Information 1 [file peerj-cs-09-1729-s001.zip › code1/inteligent_algorithm_submit/Function10/func10/pic28.jpg]

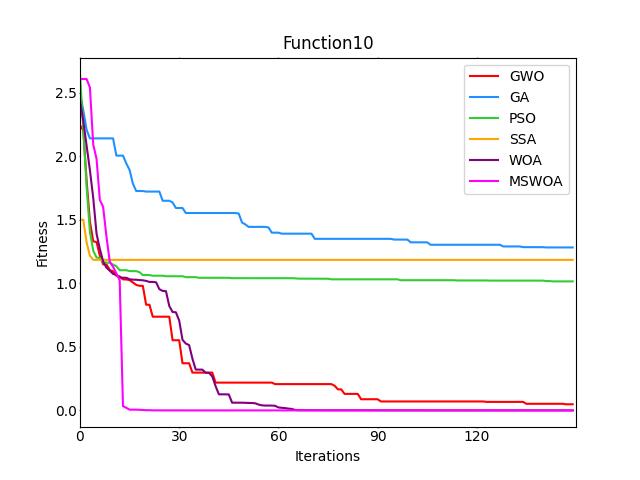

Supplement: Supplemental Information 1 [file peerj-cs-09-1729-s001.zip › code1/inteligent_algorithm_submit/Function10/func10/pic29.jpg]

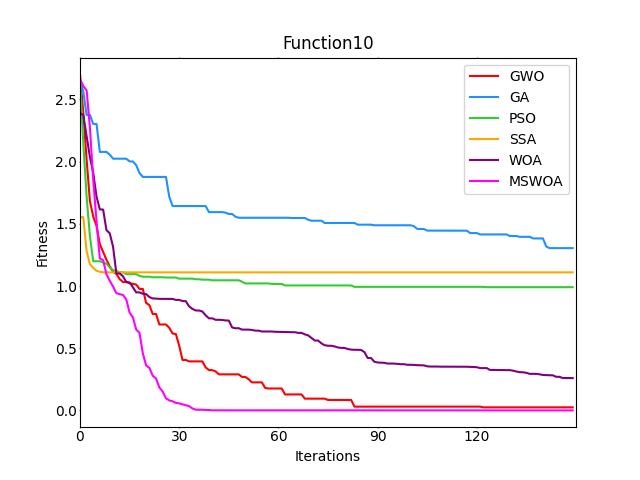

Supplement: Supplemental Information 1 [file peerj-cs-09-1729-s001.zip › code1/inteligent_algorithm_submit/Function10/func10/pic3.jpg]

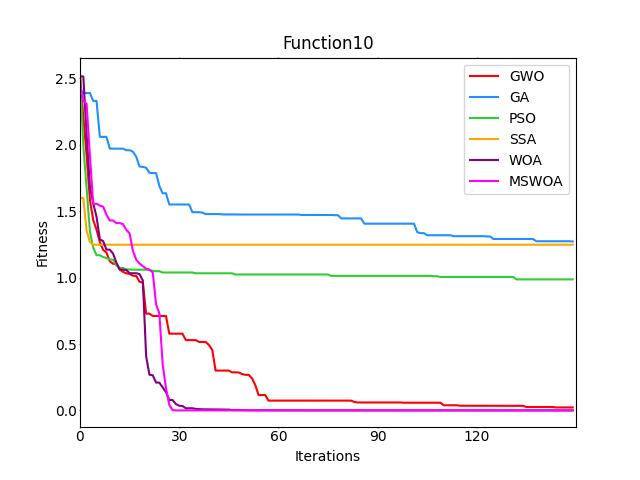

Supplement: Supplemental Information 1 [file peerj-cs-09-1729-s001.zip › code1/inteligent_algorithm_submit/Function10/func10/pic30.jpg]

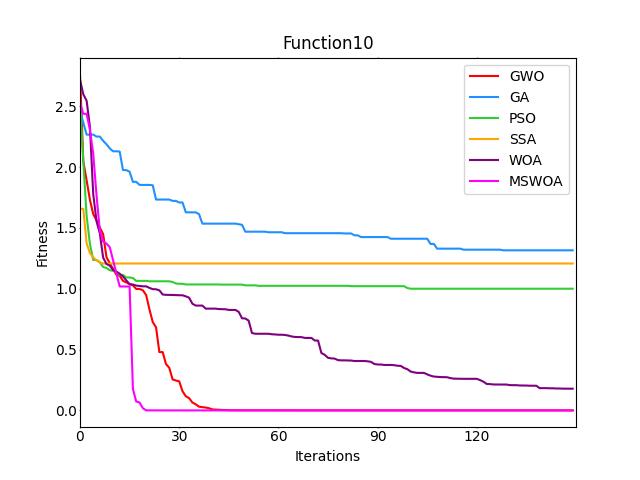

Supplement: Supplemental Information 1 [file peerj-cs-09-1729-s001.zip › code1/inteligent_algorithm_submit/Function10/func10/pic4.jpg]

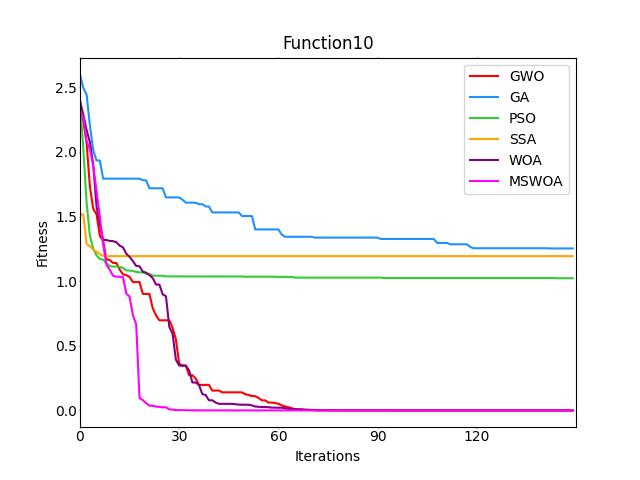

Supplement: Supplemental Information 1 [file peerj-cs-09-1729-s001.zip › code1/inteligent_algorithm_submit/Function10/func10/pic5.jpg]

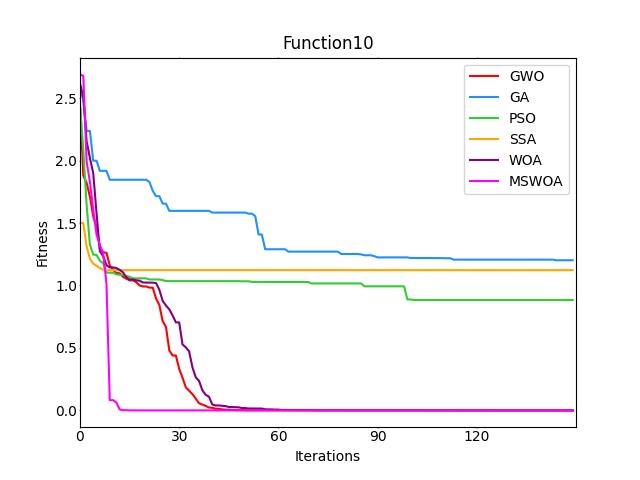

Supplement: Supplemental Information 1 [file peerj-cs-09-1729-s001.zip › code1/inteligent_algorithm_submit/Function10/func10/pic6.jpg]

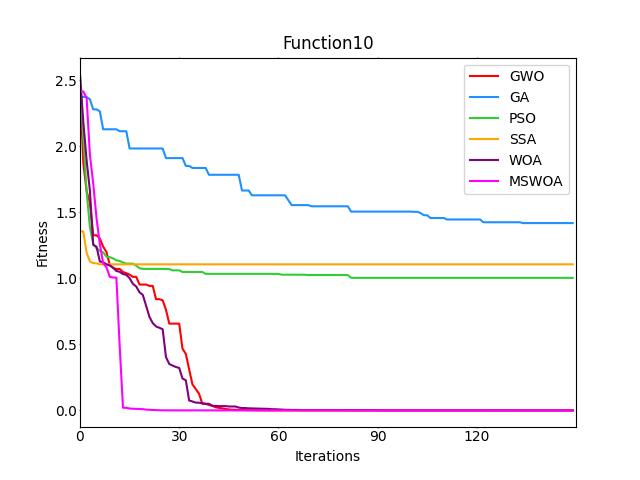

Supplement: Supplemental Information 1 [file peerj-cs-09-1729-s001.zip › code1/inteligent_algorithm_submit/Function10/func10/pic7.jpg]

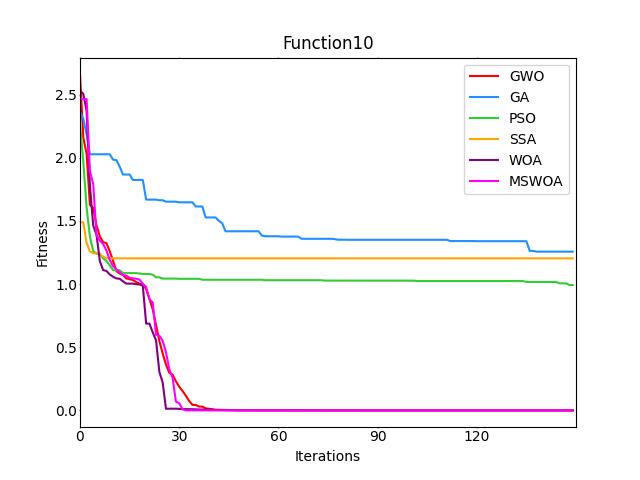

Supplement: Supplemental Information 1 [file peerj-cs-09-1729-s001.zip › code1/inteligent_algorithm_submit/Function10/func10/pic8.jpg]

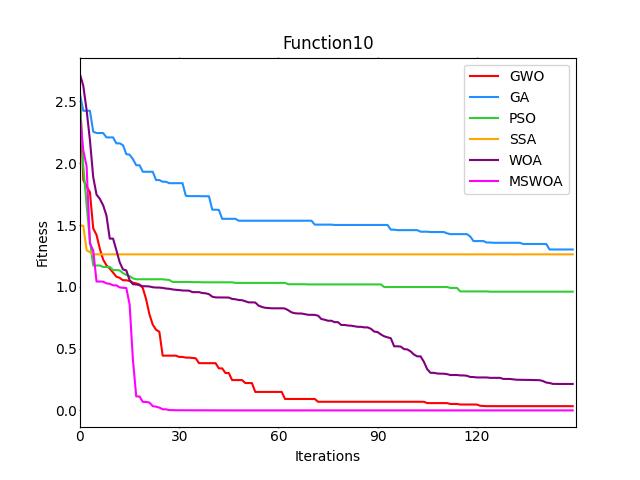

Supplement: Supplemental Information 1 [file peerj-cs-09-1729-s001.zip › code1/inteligent_algorithm_submit/Function10/func10/pic9.jpg]

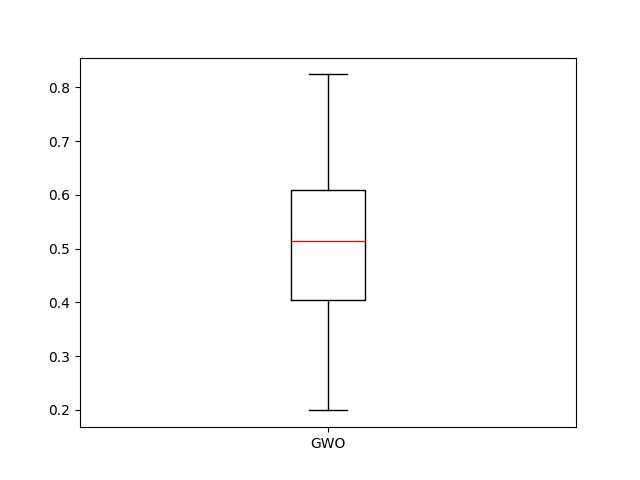

Supplement: Supplemental Information 1 [file peerj-cs-09-1729-s001.zip › code1/inteligent_algorithm_submit/Function11/boxplot_func11/pic1.jpg]

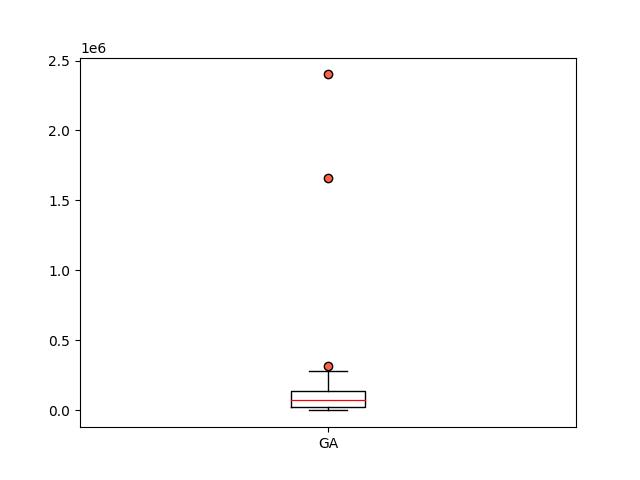

Supplement: Supplemental Information 1 [file peerj-cs-09-1729-s001.zip › code1/inteligent_algorithm_submit/Function11/boxplot_func11/pic2.jpg]

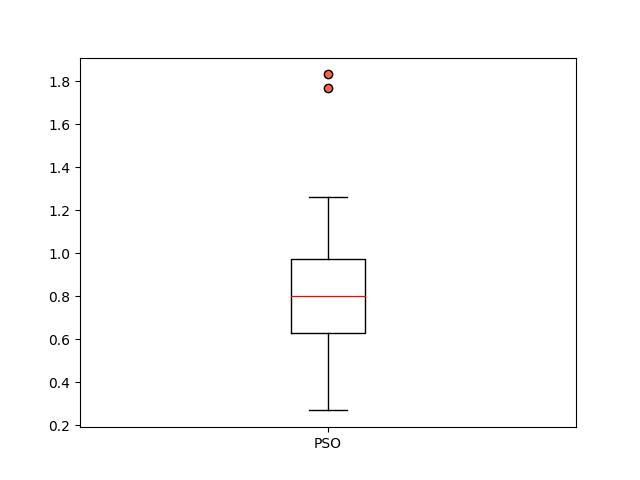

Supplement: Supplemental Information 1 [file peerj-cs-09-1729-s001.zip › code1/inteligent_algorithm_submit/Function11/boxplot_func11/pic3.jpg]

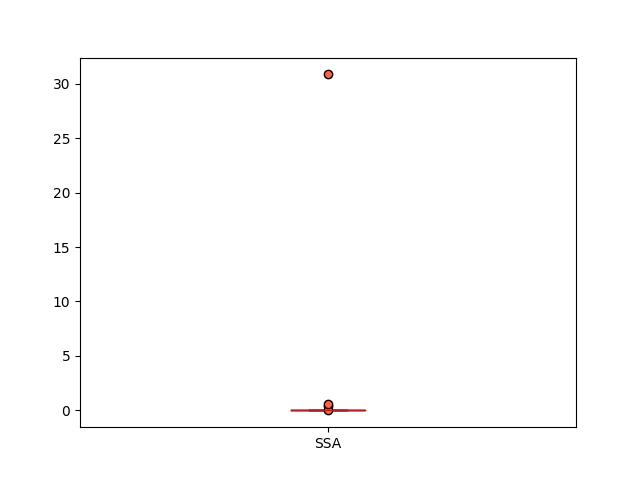

Supplement: Supplemental Information 1 [file peerj-cs-09-1729-s001.zip › code1/inteligent_algorithm_submit/Function11/boxplot_func11/pic4.jpg]

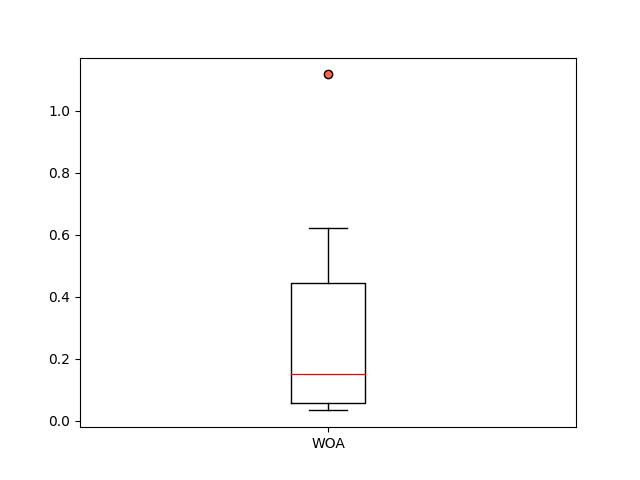

Supplement: Supplemental Information 1 [file peerj-cs-09-1729-s001.zip › code1/inteligent_algorithm_submit/Function11/boxplot_func11/pic5.jpg]

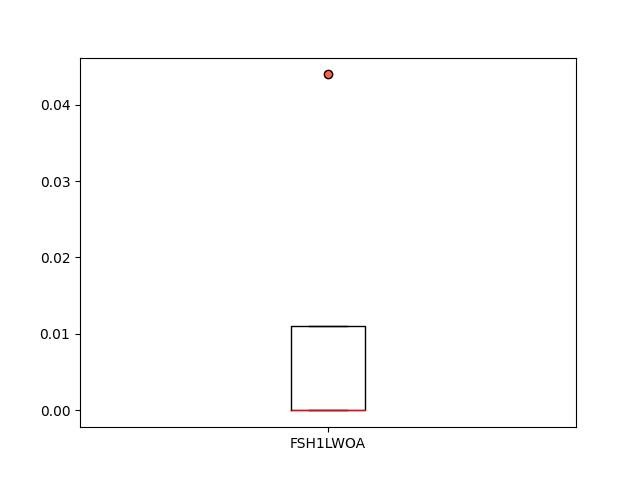

Supplement: Supplemental Information 1 [file peerj-cs-09-1729-s001.zip › code1/inteligent_algorithm_submit/Function11/boxplot_func11/pic6.jpg]

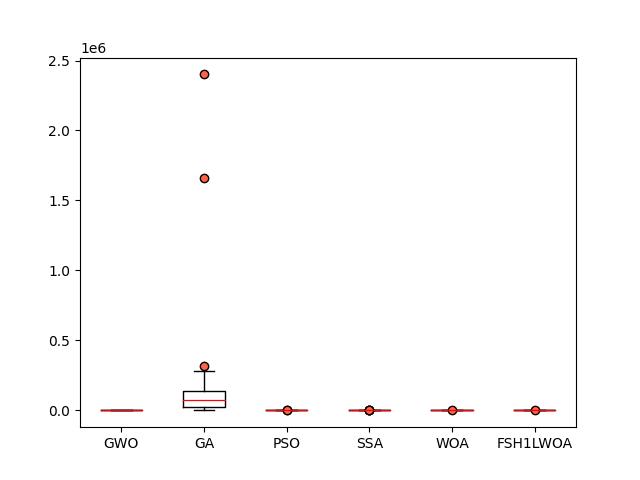

Supplement: Supplemental Information 1 [file peerj-cs-09-1729-s001.zip › code1/inteligent_algorithm_submit/Function11/boxplot_func11/pic_total.jpg]

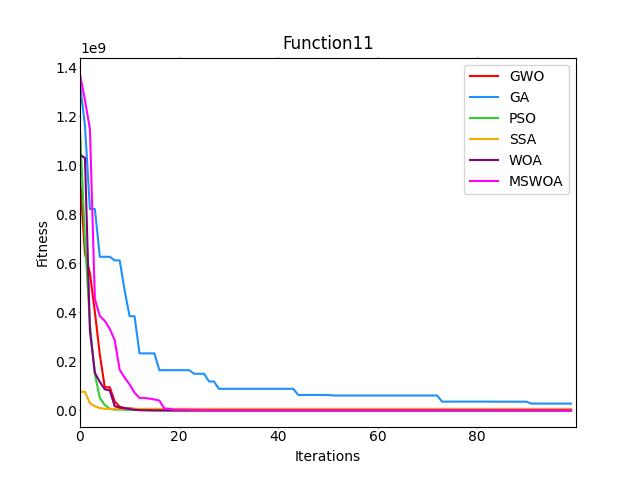

Supplement: Supplemental Information 1 [file peerj-cs-09-1729-s001.zip › code1/inteligent_algorithm_submit/Function11/func11/pic1.jpg]

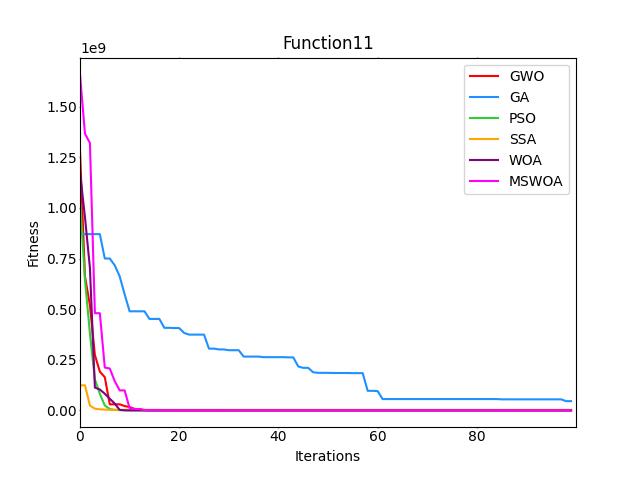

Supplement: Supplemental Information 1 [file peerj-cs-09-1729-s001.zip › code1/inteligent_algorithm_submit/Function11/func11/pic10.jpg]

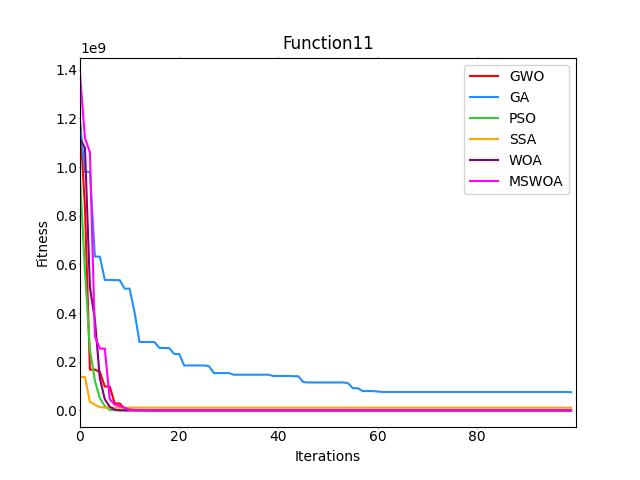

Supplement: Supplemental Information 1 [file peerj-cs-09-1729-s001.zip › code1/inteligent_algorithm_submit/Function11/func11/pic11.jpg]

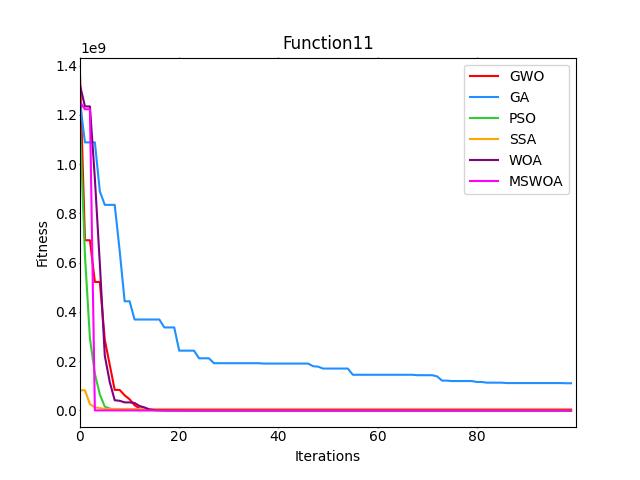

Supplement: Supplemental Information 1 [file peerj-cs-09-1729-s001.zip › code1/inteligent_algorithm_submit/Function11/func11/pic12.jpg]

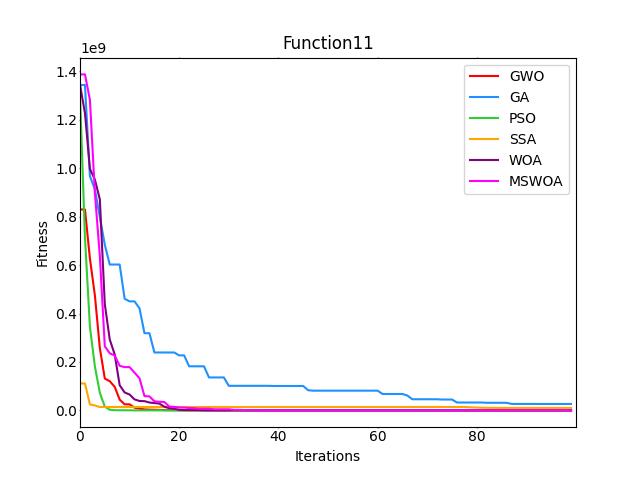

Supplement: Supplemental Information 1 [file peerj-cs-09-1729-s001.zip › code1/inteligent_algorithm_submit/Function11/func11/pic13.jpg]

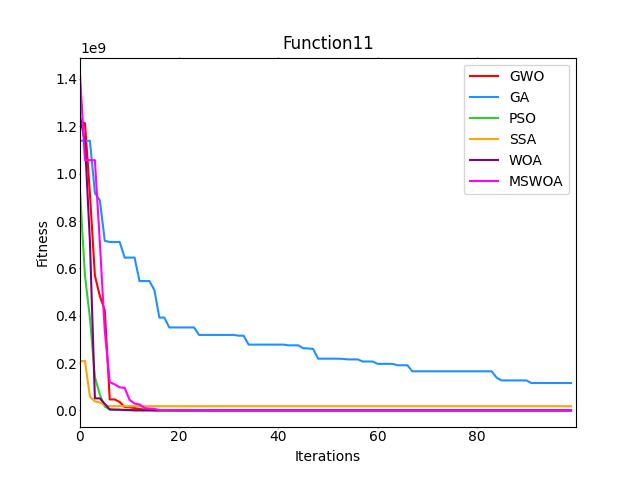

Supplement: Supplemental Information 1 [file peerj-cs-09-1729-s001.zip › code1/inteligent_algorithm_submit/Function11/func11/pic14.jpg]

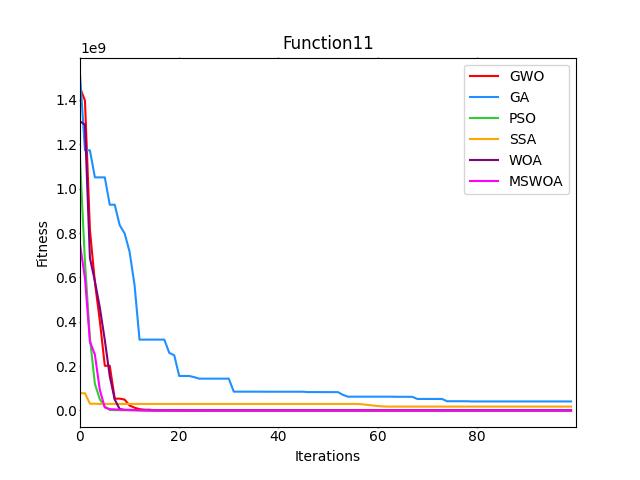

Supplement: Supplemental Information 1 [file peerj-cs-09-1729-s001.zip › code1/inteligent_algorithm_submit/Function11/func11/pic15.jpg]

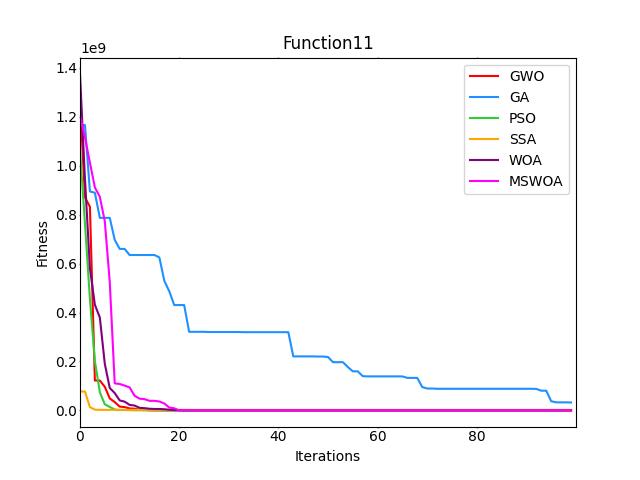

Supplement: Supplemental Information 1 [file peerj-cs-09-1729-s001.zip › code1/inteligent_algorithm_submit/Function11/func11/pic16.jpg]

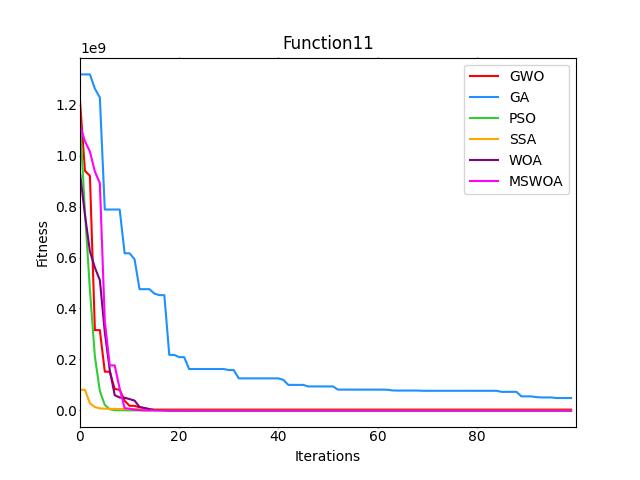

Supplement: Supplemental Information 1 [file peerj-cs-09-1729-s001.zip › code1/inteligent_algorithm_submit/Function11/func11/pic17.jpg]

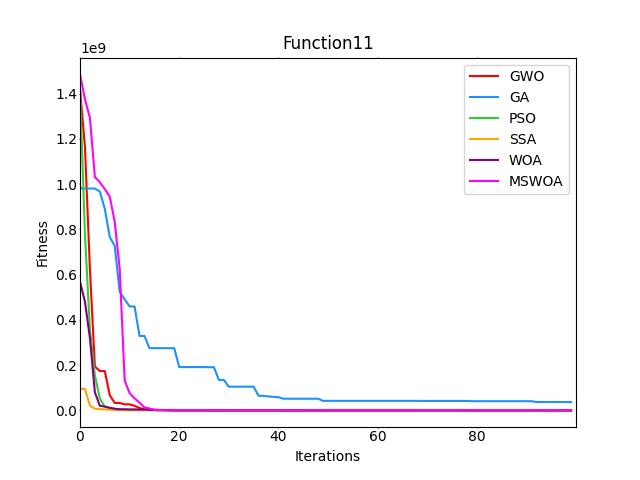

Supplement: Supplemental Information 1 [file peerj-cs-09-1729-s001.zip › code1/inteligent_algorithm_submit/Function11/func11/pic18.jpg]

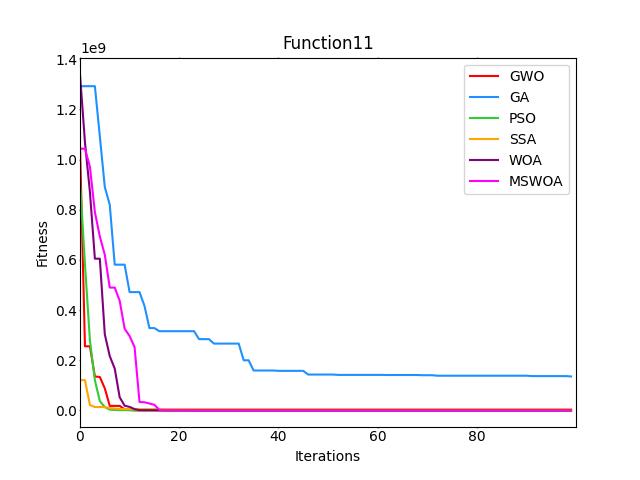

Supplement: Supplemental Information 1 [file peerj-cs-09-1729-s001.zip › code1/inteligent_algorithm_submit/Function11/func11/pic19.jpg]

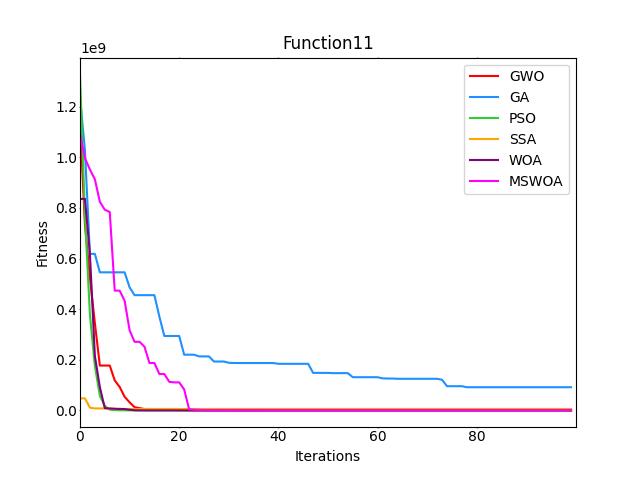

Supplement: Supplemental Information 1 [file peerj-cs-09-1729-s001.zip › code1/inteligent_algorithm_submit/Function11/func11/pic2.jpg]

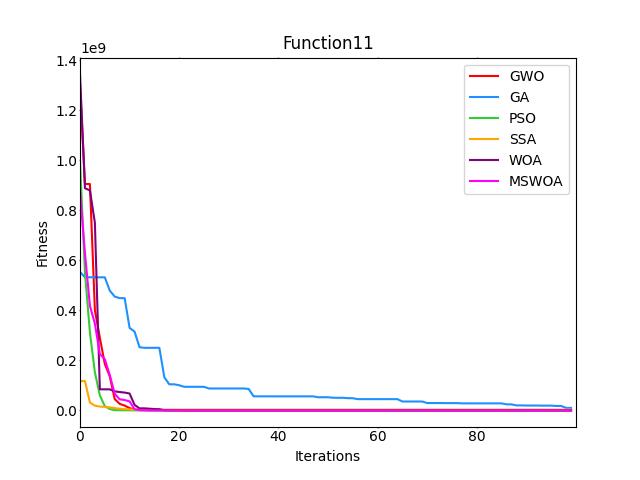

Supplement: Supplemental Information 1 [file peerj-cs-09-1729-s001.zip › code1/inteligent_algorithm_submit/Function11/func11/pic20.jpg]

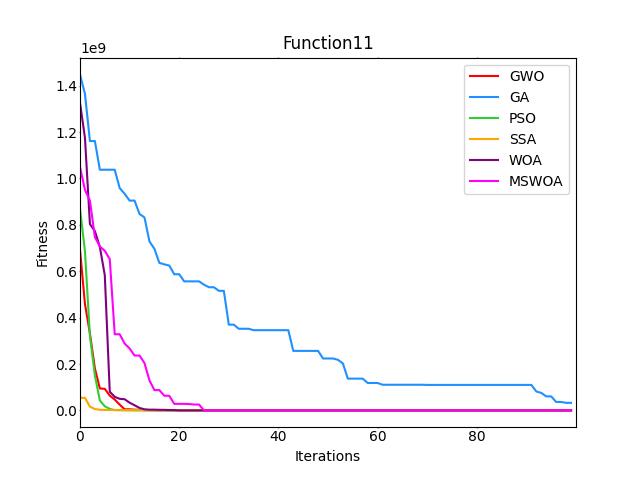

Supplement: Supplemental Information 1 [file peerj-cs-09-1729-s001.zip › code1/inteligent_algorithm_submit/Function11/func11/pic21.jpg]

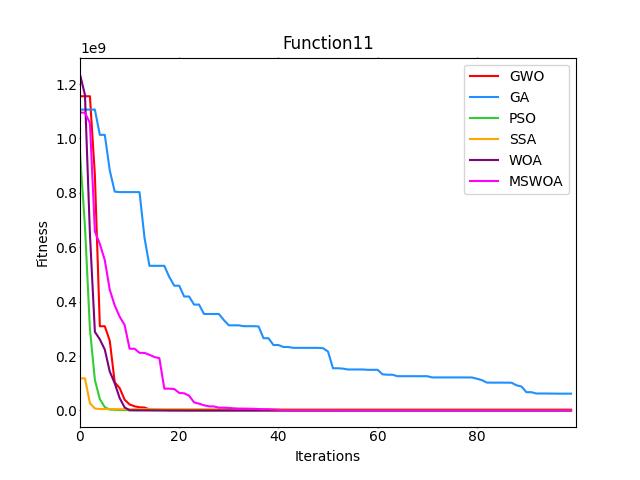

Supplement: Supplemental Information 1 [file peerj-cs-09-1729-s001.zip › code1/inteligent_algorithm_submit/Function11/func11/pic22.jpg]

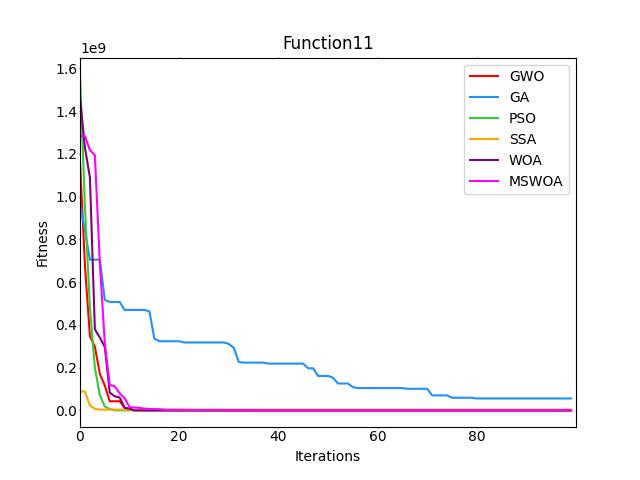

Supplement: Supplemental Information 1 [file peerj-cs-09-1729-s001.zip › code1/inteligent_algorithm_submit/Function11/func11/pic23.jpg]

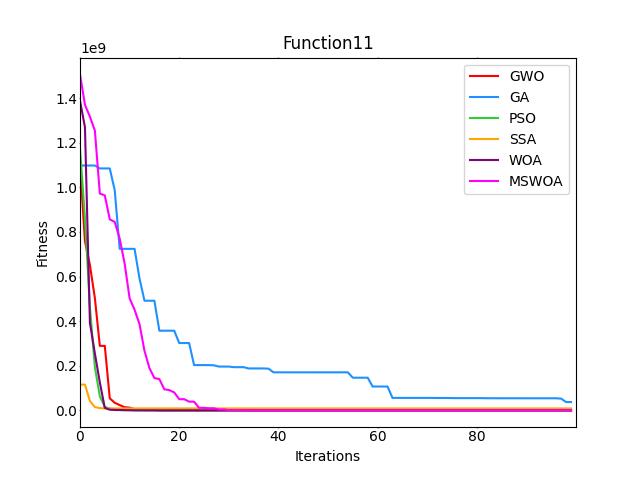

Supplement: Supplemental Information 1 [file peerj-cs-09-1729-s001.zip › code1/inteligent_algorithm_submit/Function11/func11/pic24.jpg]

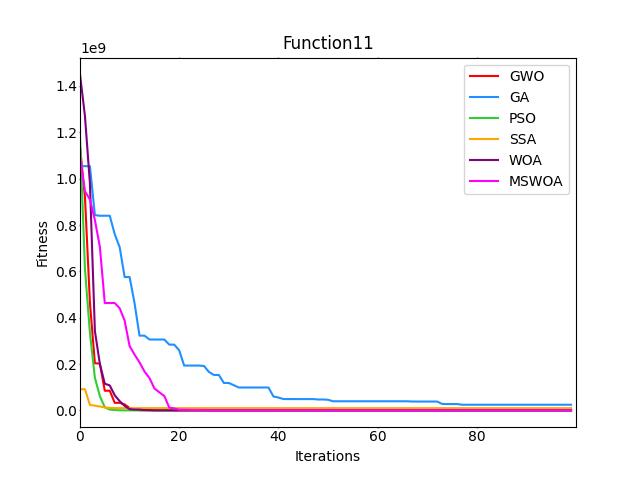

Supplement: Supplemental Information 1 [file peerj-cs-09-1729-s001.zip › code1/inteligent_algorithm_submit/Function11/func11/pic25.jpg]

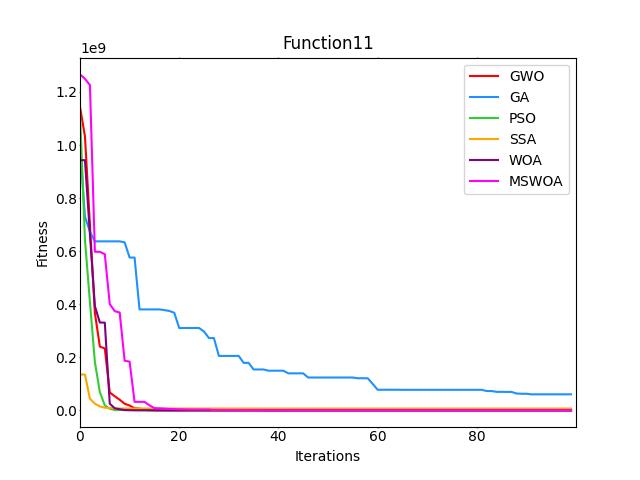

Supplement: Supplemental Information 1 [file peerj-cs-09-1729-s001.zip › code1/inteligent_algorithm_submit/Function11/func11/pic26.jpg]

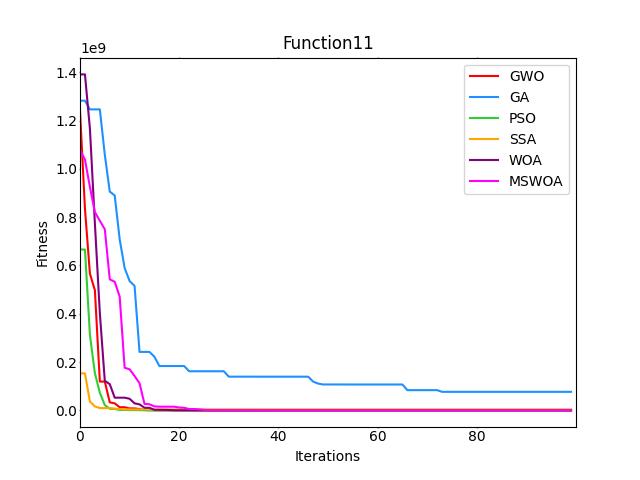

Supplement: Supplemental Information 1 [file peerj-cs-09-1729-s001.zip › code1/inteligent_algorithm_submit/Function11/func11/pic27.jpg]
